# Circulating Metabolic Factors Mediating the Effect of Obesity‐Related Indicators on Meniscal Injuries: A Mendelian Randomization Study

**DOI:** 10.1155/ijog/8056288

**Published:** 2026-02-23

**Authors:** Dong Ye, Zhiping Zhang, Jun Zhang

**Affiliations:** ^1^ Department of Orthopedics, The First Hospital of Nanchang, The Third Affiliated Hospital of Nanchang University, Nanchang, Jiangxi, China, ncsdyyy.com

**Keywords:** blood metabolites, Mendelian analysis, meniscal injuries, obesity-related indicators

## Abstract

**Background:**

Meniscal injuries and obesity are major global public health problems. The Mendelian randomization approach can overcome the limitations of traditional observational study designs with respect to confounding and reverse causation. The aims of the current study are to assess the causal relationship between obesity‐related indicators and meniscal injuries using MR analyses and to explore the potential underlying mechanism.

**Methods:**

In total, seven obesity‐related indicators and 11 circulating metabolic indicators were downloaded as instrumental variables from published genome‐wide association studies (GWAS), and the meniscal injury data from the FinnGen database was used as the outcome indicators. The causal relationships and mediating factors were analyzed using two‐sample univariate MR, multivariate MR, and intermediate MR.

**Results:**

After applying the Bonferroni correction, the IVW model indicated that five obesity‐related indicators, namely, waist circumference (*p* < 0.001, OR = 1.6333), BMI (*p* < 0.001, OR = 1.5175), body fat percentage (*p* < 0.001, OR = 1.5449), and left (*p* < 0.001, OR = 1.9432)/right (*p* < 0.001, OR = 1.9099) leg fat percentage, increased the risk of meniscal injuries, and total body bone mineral density (*p* = 0.0014, OR = 1.1098) also increased the risk of meniscal injury. The direction of MR‐identified causal relationships was consistent without horizontal pleiotropy. Multivariate and mediated MR analyses revealed that the hazardous effects of body fat percentage might be mediated by serum uric acid levels.

**Conclusions:**

Our study suggests that increases in serum uric acid levels, driven by body fat percentage, may increase the risk of meniscal injuries. We hope that these findings will provide new insights for the prevention and treatment of meniscal injury.

## 1. Introduction

The menisci, paired crescent‐shaped fibrocartilaginous discs located in the knee joint, play crucial roles in load transmission, shock absorption, joint proprioception, and nutrient supply [1, 2]. Meniscal injuries are caused by a variety of factors and are strongly associated with an increased risk of osteoarthritis (OA) [3, 4]. Epidemiological evidence has indicated that the prevalence of meniscal injuries exceeds 60% in individuals over 50 years of age. Arthroscopic partial meniscectomy is associated with an increased risk of knee OA and is a leading cause of musculoskeletal disorders in developed countries [5, 6].

Obesity is defined as excessive adipose tissue accumulation or aberrant distribution of body fat (BF) that impairs physiological function, and it has emerged as a global health burden with escalating prevalence [7]. Body mass index (BMI), a widely used anthropometric measure, is strongly associated with meniscal injuries [8]. However, BMI has critical limitations in characterizing adipose tissue biology, as it fails to reflect the distribution of fat across different body regions and cannot distinguish between lean muscle mass and fat. Therefore, the precise correlations and potential causal relationships between obesity‐related phenotypes, such as waist circumference (WC), hip circumference (HC), body fat percentage (BFP), and the left/right thigh fat ratio [9], and meniscal injuries require further investigation.

Additionally, emerging evidence indicates that cartilage injuries are prevalent in non‐weight‐bearing joints and more severe in obese people, suggesting that such injuries extend beyond mere mechanical stress, in which chronic low‐grade inflammation may play a central role [10–13]. Multiple studies have established that metabolic dysregulation (glucose, insulin, lipids, and serum uric acid) may significantly influence the development or clinical progression of OA through the regulation of proinflammatory/anti‐inflammatory and anabolic/catabolic balance, matrix remodeling, chondrocyte apoptosis and autophagy, and subchondral bone sclerosis [14–16]. These findings suggest that metabolic syndrome (MetS) may indirectly accelerate the degenerative process of meniscal tissue via systemic metabolic disorders, thereby acting as a key pathological bridge between obesity and cartilage injuries.

However, existing studies are mostly based on observational designs, making it difficult to distinguish between causal relationships and confounding effects. Additionally, large‐scale multicenter clinical trials are often time‐consuming and costly, with inherent limitations in establishing causal inference. Mendelian randomization (MR), a robust method that uses genetic variants from genome‐wide association studies (GWAS) as instrumental variables (IVs), enables causal inference between exposures and outcomes in clinical research while being unaffected by confounding factors and reverse causality [17–20]. In this study, we applied univariate, multivariate Mendelian randomization (MVMR), and intermediate MR approaches to conduct MR analyses, with the aim of investigating the causal effects and mediating roles of observed obesity‐related indicators and metabolic disorders in meniscal injuries. Sensitivity analyses were performed to evaluate the influence of methodological assumptions on the results and enhance the robustness of our findings. These results will advance the understanding of the mechanisms by which obesity‐related indicators contribute to meniscal injuries and provide a theoretical basis for developing targeted intervention strategies grounded in metabolic regulation.

## 2. Method

### 2.1. Study Design

Using the GWAS and publicly available datasets, the causal effects of obesity‐related indicators and metabolic markers on meniscal injuries were investigated, and potential mediators were explored via MR. The methods used in this study adhered to the Strengthening the Reporting of Observational Studies in Epidemiology Using Mendelian Randomization (STROBE‐MR Statement) [21]. A schematic of the study is presented in Figure 1.

Figure 1Flow chart of the MR analysis. (a) The analysis diagram of multivariate MR and the intermediary role. (b) Flow chart of analytical methods of this study.

**Figure:**
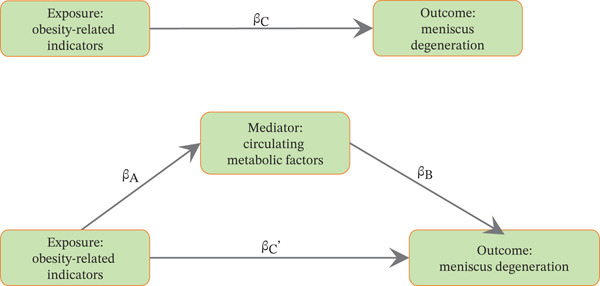
(a)

**Figure:**
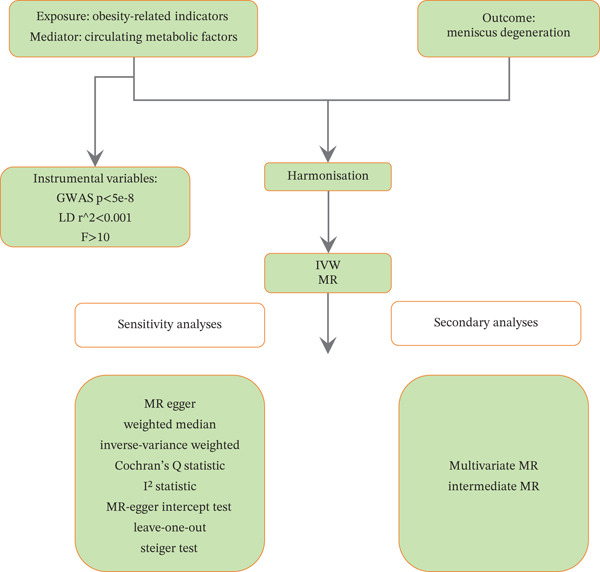
(b)

### 2.2. Data Sources of Exposures, Mediators, and Outcomes

In this study, all data sources used in this study were obtained from publicly available GWAS, with detailed provenance information provided in Supporting Information 8: Table S1 and Supporting Information 9: Table S2. The GWAS datasets underwent rigorous quality control procedures and obtained ethical approval from their respective institutional review boards, along with documented informed consent from all participants. Therefore, no additional ethics approval was required for the present study.

To comprehensively evaluate the causal relationship between obesity and meniscal injuries, we quantified adiposity using multiple anthropometric indices, such as HC, WC, waist‐to‐hip ratio (WHR), BMI, and three BF‐specific metrics: BFP and left/right leg fat percentage.

The FinnGen data is a nationwide GWAS, integrating longitudinal phenotypic data with digital health records from the National Health Registry [22]. The meniscal injury dataset is sourced from the FinnGen portal, comprising 31,840 cases and 315,115 controls. The target trait of interest, meniscal injuries, was defined using ICD10 Codes M23.0–M23.3, including all subcodes.

Circulating metabolic factor levels significantly influence OA development [14–16]. Through comprehensive literature screening, 12 candidate mediators were identified, including serum uric acid levels [23], total cholesterol levels [24], triglyceride levels [24], HDL cholesterol levels [24], LDL cholesterol levels [24], apolipoprotein A1 levels [25], apolipoprotein B levels [26], Type 1 diabetes [27], Type 2 diabetes [27], bone mineral density (BMD) [28], calcium levels [29], and serum 25‐hydroxyvitamin D levels [30]. The summary data used in this study were extracted from publicly available databases and published studies, with a focus on data from individuals of European ancestry to reduce potential bias due to population heterogeneity.

### 2.3. Selection of Genetic IVs

In the study, the criteria of IV screening for exposure were set as follows: First, the SNPs identified in the GWAS data with a *p* < 5 × 10^−8^ were retained. Second, SNPs in linkage disequilibrium (LD), with an *R* ^2^ value of less than 0.001 and a physical distance > 10,000 kb, were removed. Third, to reduce the bias from weak IVs, the *F* statistics of the SNP were also calculated, which represent the strength of the IVs, and the formula is as follows:

F=N−k−1k×R21−R2 

### 2.4. MR Analysis

Two‐sample MR was applied to evaluate the causal effects of obesity‐related indicators and circulating metabolic indicators on meniscal injuries. Specifically, when directional pleiotropy and heterogeneity are absent, the inverse variance weighted (IVW, fixed‐effect) method serves as the primary approach. If heterogeneity is detected, the IVW (random‐effects) method serves as the primary approach, and the weighted median method and the MR‐Egger regression serve as supplementary analyses [31]. We set the statistical significance threshold at a *p* value < 0.05 for UVMR. For circulating metabolic factors and obesity‐related indicators, significance thresholds were Bonferroni‐corrected to *p* < 0.0045 (0.05/11) and *p* < 0.0071 (0.05/7), respectively. In cases of directional pleiotropy, results are excluded. Finally, the direction of the causal relationship was determined using the Steiger test.

### 2.5. Sensitivity Analysis

Given the absence of a pleiotropy assumption in the MR, sensitivity analyses were conducted to validate the robustness of our findings. Three primary methods were employed: heterogeneity testing, horizontal pleiotropy testing, and leave‐one‐out analysis.

First, Cochran′s *Q* statistic was adopted to assess heterogeneity among the IVs, and the *I* ^2^ statistic further quantifies the proportion of total variation attributable to within‐IV heterogeneity. Specifically, an *I* ^2^ value ≤ 0% is defined as no heterogeneity, values between 0% and 25% indicate mild heterogeneity, 25%–50% denote moderate heterogeneity, and > 50% suggest substantial heterogeneity. Moreover, the IVW fixed‐effect model was applied to estimate the causal effect when heterogeneity was low, mild, and moderate, and the IVW random‐effects model was applied for a highly heterogeneous index. The calculation formula is as follows:

I2=Q−dlQ×100%

Second, horizontal pleiotropy was evaluated using the MR‐Egger. A statistically significant intercept (*p* < 0.05) indicated the presence of horizontal pleiotropy, and the results were removed.

### 2.6. MVMR Analysis and Mediating Effect Estimation

For the MVMR analysis, we selected exposures with significant causal associations identified in univariable MR analyses and conducted MVMR analyses using the MVMR‐IVW method, aiming to adjust for potential confounders and construct MVMR models for obesity‐related indicators, circulating metabolic factors, and meniscal injuries. Through the MVMR analysis, the direct effects of obesity‐related indicators and circulating metabolic factors on meniscal injuries were estimated, and univariable MR was conducted to obtain the effects of obesity‐related indicators on circulating metabolic factors, enabling us to estimate the indirect effects of the obesity‐related indicators → circulating metabolic factors → meniscal injuries. Using the following equations, the effect sizes and standard errors of the mediation effects were calculated:

βM=βA×βBSEM=βA×SEB2+βB×SEA2

Combining the causal stepwise regression, if both *β*_*A*_ and *β*_*B*_ are statistically significant, the indirect effect is considered significant. If either *β*_*A*_ or *β*_*B*_ is not significant, the Sobel test is applied to determine the significance of *β*_*M*_. If *β*_*M*_ is significant, the indirect effect is significant. Under the premise of a significant indirect effect, if the MR effect value *β*_*C*_ of the obesity‐related indicator in MVMR is statistically significant, the direct effect is considered significant, and there might be other mediator variables. Conversely, if *β*_*C*_ is not significant, the direct effect is deemed nonsignificant, suggesting a potential complete mediation effect. Given the complexity of mediation effects, the study focuses on discussing mediation only when there is a significant causal association between the obesity‐related indicator and the meniscal injuries and a significant causal association between the obesity‐related indicator and the mediating factor.

### 2.7. Statistical Analysis

All data analyses were performed using the “TwoSampleMR” package (version 4.3.0) in R software. Horizontal pleiotropy was evaluated using the MR‐Egger intercept method. Odds ratios (ORs), 95% confidence intervals (CIs), and *p* values were reported. For the SNPs derived from the GWAS dataset, *p* < 5 × 10^−8^ was considered statistically significant. For other statistical inferences, *p* < 0.05 was considered statistically significant.

## 3. Results

### 3.1. IV Screening

In accordance with the IV selection criteria applied in our study, SNPs with LD were removed. Following matching with the GWAS data for meniscal injuries, the SNPs of obesity‐related indicators and circulating metabolic factors were included as IVs. IVs identified via MR analysis with statistically significant *p* values (*p* < 5 × 10^−8^) are shown in Supporting Information 10: Table S3 and Supporting Information 11: Table S4. The *F* statistic for these IVs is greater than 10, indicating that the selected SNPs have a strong impact and that potential bias is limited.

### 3.2. MR Causal Effect Estimates

MR analyses were conducted using three models: MR‐Egger, weighted median, and IVW. The results of the IVW model were used as the screening criterion. Causal effect estimates from these three models are presented in Supporting Information 12: Table S5 and Supporting Information 13: Table S6.

**Figure 2:**
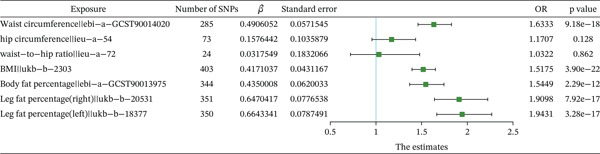
Forest plot of the IVW model for the MR of obesity‐related indicators on meniscal injuries.

Scatter plots of SNP‐based MR analysis between obesity‐related indicators and meniscal injuries are shown in Supporting Information 1: Figure S1. The results demonstrated that the fitted curves of the three models exhibited consistent directions, with the slopes of most models indicating relative consistency. Additionally, the intercept of the IVW model was close to 0, suggesting no substantial horizontal pleiotropy. Combining the results from the heterogeneous analyses, the results of the IVW model are presented in Supporting Information 14: Table S7 and Supporting Information 15: Table S8 and Figure 2 for obesity‐related indicators. The IVW model results indicated a significant causal relationship (*p* < 0071) between obesity‐related indicators and meniscal injuries, such as WC (*p* < 0.01, OR = 1.6333), BMI (*p* < 0.01, OR = 1.5175), BFP (*p* < 0.01, OR = 1.5449), and left (*p* < 0.01, OR = 1.9432)/right (*p* < 0.01, OR = 1.9099) leg fat percentage. The causal direction from obesity‐related indicators to meniscal injuries was correct according to the MR Steiger test for directionality (Supporting Information 16: Table S9).

**Figure 3:**
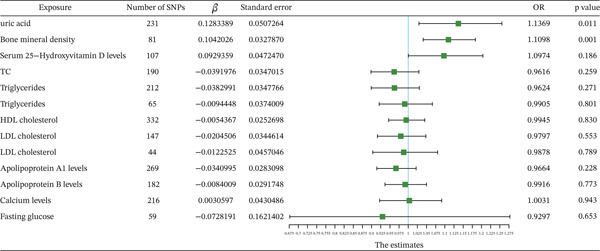
Forest plot of the IVW model for the MR of circulating metabolic factors on meniscal injuries.

Scatter plots of the SNP‐based MR analysis between circulating metabolic indicators and meniscal injuries are shown in Supporting Information 2: Figure S2. The results demonstrated that the fitted curves of the three models exhibited consistent directions, with the slopes of most models exhibiting relative consistency. The intercept of the IVW model was close to 0, suggesting no substantial horizontal pleiotropy. Combining the results from the heterogeneous analyses, the results of the IVW model are presented in Supporting Information 17: Table S10 and Figure 3 for circulating metabolic indicators. The IVW model results indicated a significant causal relationship (*p* < 0.0045) between circulating metabolic indicators and meniscal injuries, such as BMD (*p* = 0.00148, OR = 1.1098). The causal direction from circulating metabolic indicators to meniscal injuries was correct according to the MR Steiger test for directionality (Supporting Information 18: Table S11).

### 3.3. Sensitivity Analysis

#### 3.3.1. Obesity‐Related Indicators

The *I* ^2^ statistical results indicated that the heterogeneity ratio of the MR findings between most obesity‐related indicators and meniscal injuries was low and mild (*I* ^2^ < 50*%*, Supporting Information 19: Table S12). Funnel plots of the IVs of obesity‐related indicators are presented in Supporting Information 3: Figure S3. Most of the data points in this analysis were distributed on both sides of the blue indicator line of the IVW model, indicating the presence of a small degree of publication bias.

According to the MR‐Egger regression results, horizontal pleiotropy did not affect the causal inference between obesity‐related indicators and meniscal injuries (Supporting Information 20: Table S13).

The leave‐one‐out sensitivity analysis revealed that no SNP significantly affected these results, validating the stability of our findings (Supporting Information 4: Figure S4).

#### 3.3.2. Circulating Metabolic Indicators

The *I* ^2^ statistical results indicated that the heterogeneity ratio of the MR findings between circulating metabolic indicators and meniscal injuries was low and mild (*I* ^2^ < 50*%*, Supporting Information 21: Table S14). Funnel plots of the IVs of circulating metabolic indicators are presented in Supporting Information 5: Figure S5. Most of the data points in this analysis were distributed on both sides of the blue indicator line of the IVW model, indicating the presence of a small degree of publication bias.

According to the MR‐Egger regression results, horizontal pleiotropy did not affect the causal inference between circulating metabolic indicators and meniscal injuries (Supporting Information 22: Table S15).

The leave‐one‐out sensitivity analysis revealed that no SNP significantly affected these results, validating the stability of our findings (Supporting Information 6: Figure S6 and Supporting Information 7: Figure S7).

### 3.4. MVMR Analysis

#### 3.4.1. MR Causal Effects of Obesity‐Related Indicators on Circulating Metabolic Factors

On the basis of the above results, we identified several obesity‐related indicators and circulating metabolic indicators that have a significant causal relationship with meniscal injuries. According to the MR‐Egger regression results (Supporting Information 23: Table S16), a statistically significant intercept (*p* < 0.05) indicated the presence of horizontal pleiotropy, and the results were removed from the subsequent studies. The *I* ^2^ statistical results indicated that the heterogeneity ratio of the MR findings between most obesity‐related indicators and circulating metabolic indicators was high (*I* ^2^ > 50*%*, Supporting Information 24: Table S17). The causal direction from obesity‐related indicators to circulating metabolic factors was correct according to the MR Steiger test for directionality (Supporting Information 25: Table S18). IVs identified via MR analysis with statistically significant *p* values (*p* < 5 × 10^−8^) are shown in Supporting Information 26: Table S19. The *F* statistic for these IVs is greater than 10, indicating that the selected SNPs have a strong impact and that potential bias is limited.

**Figure 4:**
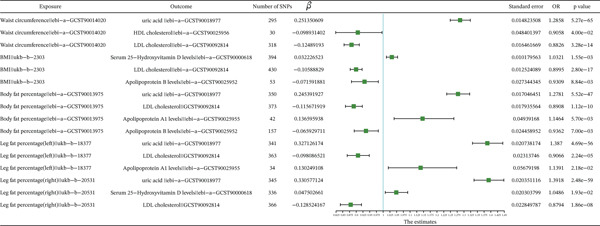
Forest plot of the IVW model for the MR of obesity‐related indicators on circulating metabolic factors.

MR analysis of obesity‐related indicators and circulating metabolic indicators was conducted using three models: MR‐Egger, weighted median, and IVW. The results of the IVW model were used as the screening criterion. Causal effect estimates from these three models are presented in Supporting Information 27: Table S20, and the significant causal relationship results are shown in Figure 4 and Supporting Information 28: Table S21 and Supporting Information 29: Table S22. The IVW model results indicated that uric acid (*p* < 0.01, OR = 1.2858), HDL cholesterol (*p* < 0.01, OR = 0.9058), and LDL cholesterol (*p* < 0.01, OR = 0.8826) were significantly related to WC; serum 25‐hydroxyvitamin D levels (*p* < 0.01, OR = 1.0321), apolipoprotein B levels (*p* < 0.01, OR = 0.8995), and LDL cholesterol (*p* < 0.01, OR = 0.9309) were significantly related to BMI; uric acid (*p* < 0.01, OR = 1.2781), LDL cholesterol (*p* < 0.01, OR = 0.8908), apolipoprotein A1 levels (*p* < 0.01, OR = 1.1464), and apolipoprotein A1 levels (*p* < 0.01, OR = 0.9362) were significantly related to BFP; uric acid (*p* < 0.01, OR = 1.3870), LDL cholesterol (*p* < 0.01, OR = 0.9066), and apolipoprotein A1 levels (*p* < 0.01, OR = 1.1391) were significantly related to left leg fat percentage; and uric acid (*p* < 0.01, OR = 1.3918), LDL cholesterol (*p* < 0.01, OR = 0.8794), and serum 25‐hydroxyvitamin D levels (*p* < 0.01, OR = 1.0486) were significantly related to right leg fat percentage.

#### 3.4.2. MVMR Analysis

These significant differences between obesity‐related indicators and circulating metabolic indicators were entered into the model for exposure, and a separate MVMR analysis was conducted for meniscal injuries. We constructed 16 significant MVMR models for evaluating the effects of circulating metabolic‐mediated obesity‐related indicators on meniscal injuries (Table 1). The results reveal that the causal association between uric acid and meniscal injuries was significant in Model 7 (*p* < 0.05), whereas no other causal associations were significant (*p* > 0.05).

**Table 1:** Results of the MVMR analysis of the effect of obesity‐related indicators and circulating metabolic indicators on meniscal injuries.

Model	Exposure	ID	*β*	Standard error	*p* value
Model 1	Waist circumference	ebi‐a‐GCST90014020	0.507367	0.0768403	4.03e − 11
	Uric acid	ebi‐a‐GCST90018977	0.0774924	0.0424616	0.0680013
Model 2	Waist circumference	ebi‐a‐GCST90014020	0.5579448	0.1220562	4.85e − 06
	HDL cholesterol	ebi‐a‐GCST90025956	−0.01452	0.0334353	0.6640939
Model 3	Waist circumference	ebi‐a‐GCST90014020	0.539504	0.0722802	8.39e − 14
	LDL cholesterol	ebi‐a‐GCST90092814	−0.012804	0.0570114	0.8222971
Model 4	BMI	ukb‐b‐2303	0.37814	0.0513314	1.75e − 13
	Serum 25‐hydroxyvitamin D levels	ebi‐a‐GCST90000618	0.0921848	0.069614	0.1854272
Model 5	BMI	ukb‐b‐2303	0.4182544	0.0510219	2.45e − 16
	LDL cholesterol	ebi‐a‐GCST90092814	−0.002871	0.0542566	0.9577923
Model 6	BMI	ukb‐b‐2303	0.4109923	0.0699759	4.27e − 09
	Apolipoprotein B levels	ebi‐a‐GCST90025952	0.0215536	0.0336189	0.5214475
Model 7	Body fat percentage	ebi‐a‐GCST90013975	0.490764	0.0869745	1.67e − 08
	Uric acid	ebi‐a‐GCST90018977	0.0907771	0.0453359	0.0452499
Model 8	Body fat percentage	ebi‐a‐GCST90013975	0.4770329	0.081987	5.94e − 09
	LDL cholesterol	ebi‐a‐GCST90092814	−0.025833	0.0589433	0.6611953
Model 9	Body fat percentage	ebi‐a‐GCST90013975	0.691562	0.122647	1.71e − 08
	Apolipoprotein A1 levels	ebi‐a‐GCST90025955	−0.029342	0.036628	0.4230881
Model 10	Body fat percentage	ebi‐a‐GCST90013975	0.6145557	0.1117915	3.86e − 08
	Apolipoprotein B levels	ebi‐a‐GCST90025952	0.0251816	0.0357104	0.4807093
Model 11	Leg fat percentage (right)	ukb‐b‐20531	0.7276057	0.1095574	3.11e − 11
	Uric acid	ebi‐a‐GCST90018977	0.0766013	0.0433053	0.0769159
Model 12	Leg fat percentage (right)	ukb‐b‐20531	0.6208084	0.1014955	9.56e − 10
	Serum 25‐hydroxyvitamin D levels	ebi‐a‐GCST90000618	0.1053012	0.0723335	0.1454551
Model 13	Leg fat percentage (right)	ukb‐b‐20531	0.6281838	0.0957654	5.39e − 11
	LDL cholesterol	ebi‐a‐GCST90092814	−0.069767	0.0493243	0.1572304
Model 14	Leg fat percentage (left)	ukb‐b‐18377	0.7249688	0.1147363	2.64e − 10
	Uric acid	ebi‐a‐GCST90018977	0.0432716	0.06181	0.4838814
Model 15	Leg fat percentage (left)	ukb‐b‐18377	0.6778478	0.1038623	6.74e − 11
	LDL cholesterol	ebi‐a‐GCST90092814	−0.011995	0.0584046	0.8372831
Model 16	Leg fat percentage (left)	ukb‐b‐18377	1.1482766	0.239017	1.55e − 06
	Apolipoprotein A1 levels	ebi‐a‐GCST90025955	−0.039815	0.034614	0.2500418

### 3.5. Mediation Effect Analysis

In the MVMR analysis, the mediation effect was evaluated in models exhibiting significant causal relationships between circulating metabolic indicators and meniscal injuries. To detect whether the mediating effects were significant for the remaining models, the Sobel test was applied and the mediation effect was also assessed in those models. The results indicated that, among all effect models, only Model 7 showed a significant effect between uric acid concentration and meniscal injuries, while no significant mediating effects were observed in the remaining models via the Sobel test (Supporting Information 30: Table S23). Given the significant causal relationship (*p* < 0.05) between obesity‐related indicators and meniscal injuries in Model 7, Model 7 may represent a partial mediation and other potential mediators might exist, as shown in Supporting Information 31: Table S24.

## 4. Discussion

Obesity, metabolic disorders, and meniscal injuries have been reported as global health concerns, with obesity commonly recognized as a risk factor for joint damage and metabolic disorders [32]. The aim of this study was to investigate the causal relationships between obesity‐related indicators and meniscal injuries, as well as potential metabolic mediators using genetic variants. Our findings suggest that WC, BMI, BFP, and left/right leg fat percentage, along with BMD, may contribute to the development of meniscal injuries. Additionally, the evidence for causal relationships between serum uric acid levels, HDL cholesterol levels, LDL cholesterol levels, triglyceride levels, serum 25‐hydroxyvitamin D levels, total cholesterol levels, calcium levels, Type 2 diabetes, Type 1 diabetes, apolipoprotein B levels, apolipoprotein A1 levels, and meniscal injuries remains uncertain; this may be due to insufficient sample size or number of SNPs, as well as methodological limitations. MVMR and mediation analyses further elucidated the mediating roles of established obesity risk factors and metabolic factors in the context of meniscal injuries.

On the basis of a meta‐analysis of observational studies, Lee et al. reported that high BMI was not associated with the risk of meniscal injury [33]. However, subsequent MR analysis revealed a causal relationship between high BMI and meniscal injury. This discrepancy may stem from the limitations of BMI in assessing BF, specifically its inability to reflect regional fat distribution or distinguish between lean muscle mass and adipose tissue [34]. Second, when sex differences were considered, the relationship between BMI and health outcomes increased in complexity. Therefore, we validated our findings using alternative measures of obesity. In their observational study, Tekaya et al. reported that WC was significantly associated with the radiographic severity of OA [35]. Additional research has shown that in adults with or at risk of knee OA, increased WC increases the risk of future functional impairment [36]. Furthermore, the basal metabolic rate and leg and whole BF mass influence the proteoglycan content and cartilage thickness, thereby promoting the onset and progression of knee OA and increasing disease risk [37–39]. Our genetic prediction results align with these observations; however, current research on joint degenerative diseases focuses primarily on articular cartilage and subchondral bone, with limited attention given to meniscal injuries. Notably, Goebel et al. proposed that meniscal injury may act as a precursor to OA, given its close association with the tibiofemoral OA process [40]. Therefore, we conducted the first comprehensive two‐sample MR analysis, MVMR, and intermediate MR to evaluate the causal relationship between obesity‐related indicators and meniscal injuries. Our findings confirmed that obesity is a significant risk factor for meniscal injuries and revealed causal associations between WC, BFP, BMI, and left/right leg fat percentage and meniscal injuries, while no significant causal associations were observed in the HC and WHR with meniscal injuries. This is consistent with previous findings, and Wang et al. found that genetic components in OA had a significant correlation with obesity‐related traits, except WHR, which was consistent with our research results [38]. According to this result, we consider that HC may also reflect the development of muscles around the hip joint, and there may be some factors that weaken the impact of biomechanical load. Of course, this does not exclude other possibilities. Further research can explore this phenomenon.

The results from the IVW model indicated that BMD may be causally associated with meniscal injuries, potentially significantly increasing the risk of meniscal injury. Previous MR research has indicated a positive causal relationship between BMD and OA risk [41]. Furthermore, multicenter observational studies revealed greater tibial cortical bone plate BMD values in patients with meniscal extrusion Grades 2/3 [42]. Our MR findings further strengthen the evidence for a potential causal mechanism linking bone mineral concentration and meniscal injuries. In addition, recent studies have shown that higher BMD can contribute to increased mechanical loading on the joints [43]. This increased loading leads to significant remodeling of the subchondral bone, often resulting in sclerosis, which is the hardening or stiffening of the bone. As the subchondral bone becomes sclerotic, it loses its normal cushioning function, leading to greater stress on the overlying cartilage. Over time, increased stress can accelerate the degradation of the cartilage [44]. Quantitative assessment of BMD may be essential for understanding the pathophysiology of meniscal injury and OA and holds promise as a biomarker for monitoring disease progression.

Further MVMR and mediating MR analyses revealed that serum uric acid may mediate the effects of BFP on meniscal injuries, suggesting that BFP may be a risk factor for meniscal injuries through the partial effects of uric acid. Recently, owing to the high prevalence of hyperuricemia, numerous clinical connections between SUA and musculoskeletal disorders such as sarcopenia, OA, rheumatoid arthritis, intervertebral disc injuries, and osteoporosis have been identified [45–48]. Long‐term exposure to elevated uric acid levels may lead to an increase in MMP‐3 and MSU deposition with an associated inflammatory burden, eventually resulting in cartilage damage, potentially leading to OA [23]. Lee et al. demonstrated that MSU crystals, together with IL‐1*β*, stimulate COX‐2 and PGE2 protein expression in human chondrocytes, thereby potentially amplifying inflammation and enhancing osteoclastogenesis [49]. However, the relationship between uric acid levels and meniscal injuries has not yet been reported. Thus, we speculate that it seems more likely that in a high uric acid and MSU crystal environment, proinflammatory cytokines can stimulate meniscus catabolic activities and lead to meniscal injuries through suppression of proteoglycan synthesis and production of MMPs. In recent years, numerous epidemiological investigations have studied the relationship between fat distribution and SUA levels. Li et al. reported that BFP was an independent risk factor for SUA and that the accumulation of BFP might contribute to elevated SUA levels in normal‐weight and lean individuals [50]. Our study primarily demonstrated that SUA may serve as a partial mediator in driving meniscal injuries in the context of excessive accumulation of BFP. In addition, no other causal associations were significant, and no significant mediating effects were observed in the remaining models, potentially due to insufficient statistical power and limited ethnic diversity. In future research, we should employ more advanced methods and encompass more extensive and diverse populations to bolster the reliability of the findings.

In conclusion, our MR analysis revealed a significant causal association between obesity‐related indicators and meniscal injuries. Moreover, Steiger′s directionality tests supported the causal effect direction from obesity‐related indicators to meniscal injuries. These findings provide initial evidence that BFP and SUA may be involved in the pathophysiology of meniscal injuries.

However, our study has limitations. First, data from European populations were used in our analyses, which restricts the broader applicability of our findings, and future GWAS data from other ethnic populations will enable further investigation into the relationship between obesity and meniscal injuries. Second, heterogeneity was observed among the studies. However, this issue was effectively addressed by employing the IVW random‐effects method as our primary approach and successfully controlling for pooled heterogeneity. Third, owing to the limitations of GWAS summary data and the lack of individual‐specific indicators, meaningful subgroup analyses could not be conducted. Finally, although sensitivity tests were employed to validate our results, we cannot completely eliminate the possibility of pleiotropy, which could confound our interpretative framework for various reasons. In future research, we should employ more advanced methods to bolster the reliability of the findings and encompass more extensive and diverse populations, incorporating individuals with varied ancestries and cultural backgrounds. Timely experimentation and clinical research validation are necessary to validate our findings and determine their potential clinical utility.

## 5. Conclusion

In this study, for the first time, we employed genetic variants as IVs to evaluate the causal effects and mediating factors of obesity‐related indicators on the risk of meniscal injuries. Our study provides convincing evidence of a genetic causal relationship between obesity‐related indicators and meniscal injuries. Moreover, through MVMR and intermediate MR, we found that SUA may serve as a partial mediator in driving meniscal injuries in the context of excessive accumulation of BFP. However, these findings require further validation and investigation in large‐scale longitudinal studies or randomized controlled trials. In addition, elucidation of the potential mechanisms underlying these observed associations may provide valuable insights into potential therapeutic targets.

**Nomenclature**
OA: osteoarthritis
GWAS: genome‐wide association study
BMD: bone mineral density
MR: Mendelian randomization
MVMR: multivariable Mendelian randomization
SNP: single‐nucleotide polymorphism
IVW: inverse variance weighted
LD: linkage disequilibrium
OR: odds ratio
CIs: confidence intervals
SUA: serum uric acid
MetS: metabolic syndrome
HC: hip circumference
WC: waist circumference
WHR: waist‐to‐hip ratio

## Author Contributions

Jun Zhang, Zhiping Zhang, and Dong Ye designed the study. Jun Zhang and Dong Ye conducted the analysis. Jun Zhang and Zhiping Zhang wrote the manuscript and had primary responsibility for the final content.

## Funding

This study was funded by the Health Commission of Jiangxi Province (Grant Number: 202211611) and the Third Affiliated Hospital of Nanchang University, Technology and Innovation Seed Fund (Grant Number: 202502003).

## Disclosure

All authors read and approved the final manuscript.

## Ethics Statement

The authors have nothing to report.

## Consent

The authors have nothing to report.

## Conflicts of Interest

The authors declare no conflicts of interest.

## Supporting Information

Additional supporting information can be found online in the Supporting Information section.

## Supporting information


**Supporting Information 1** Figure S1: Scatter plot of correlation between obesity‐related indicators and meniscal injuries. (A) Scatter plot for waist circumference with meniscal injuries. (B) Scatter plot for hip circumference with meniscal injuries. (C) Scatter plot for waist‐to‐hip ratio with meniscal injuries. (D) Scatter plot for BMI with meniscal injuries. (E) Scatter plot for body fat percentage with meniscal injuries. (F) Scatter plot for leg fat percentage (right) with meniscal injuries. (G) Scatter plot for leg fat percentage (left) with meniscal injuries.

**Supporting Information 2** Figure S2: Scatter plot of correlation between circulating metabolic factors and meniscal injuries. (A) Scatter plot for uric acid with meniscal injuries. (B) Scatter plot for bone mineral density with meniscal injuries. (C) Scatter plot for serum 25‐hydroxyvitamin D levels with meniscal injuries. (D) Scatter plot for TC with meniscal injuries. (E) Scatter plot for triglycerides (ebi‐aGCST90018975) with meniscal injuries. (F) Scatter plot for triglycerides (ebi‐aGCST90092992) with meniscal injuries. (G) Scatter plot for HDL cholesterol with meniscal injuries. (H) Scatter plot for LDL cholesterol (ebi‐a‐GCST90018961) with meniscal injuries. (I) Scatter plot for LDL cholesterol (ebia‐GCST90092814) with meniscal injuries. (J) Scatter plot for apolipoprotein A1 levels with meniscal injuries. (K) Scatter plot for apolipoprotein B levels with meniscal injuries. (L) Scatter plot for fasting glucose with meniscal injuries. (M) Scatter plot for calcium levels with meniscal injuries.

**Supporting Information 3** Figure S3: Funnel plot of heterogeneity test for MR of obesity‐related indicators on meniscal injuries. (A) Funnel plot for waist circumference with meniscal injuries. (B) Funnel plot for hip circumference with meniscal injuries. (C) Funnel plot for waist‐to‐hip ratio with meniscal injuries. (D) Funnel plot for BMI with meniscal injuries. (E) Funnel plot for body fat percentage with meniscal injuries. (F) Funnel plot for leg fat percentage (right) with meniscal injuries. (G) Funnel plot for leg fat percentage (left) with meniscal injuries.

**Supporting Information 4** Figure S4: Leave‐one‐out plots to visualize the causal effects of obesity‐related indicators on meniscal injuries. (A) Leave‐one‐out analysis for waist circumference with meniscal injuries. (B) Leave‐one‐out analysis for hip circumference with meniscal injuries. (C) Leave‐one‐out analysis for waist‐to‐hip ratio with meniscal injuries. (D) Leave‐one‐out analysis for BMI with meniscal injuries. (E) Leave‐one‐out analysis for body fat percentage with meniscal injuries. (F) Leave‐one‐out analysis for leg fat percentage (right) with meniscal injuries. (G) Leave‐one‐out analysis for leg fat percentage (left) with meniscal injuries.

**Supporting Information 5** Figure S5: Funnel plot of heterogeneity test for MR of circulating metabolic factors on meniscal injuries. (A) Funnel plot for uric acid with meniscal injuries. (B) Funnel plot for bone mineral density with meniscal injuries. (C) Funnel plot for serum 25‐hydroxyvitamin D levels with meniscal injuries. (D) Funnel plot for TC with meniscal injuries. (E) Funnel plot for triglycerides (ebi‐aGCST90018975) with meniscal injuries. (F) Funnel plot for triglycerides (ebi‐aGCST90092992) with meniscal injuries. (G) Funnel plot for HDL cholesterol with meniscal injuries. (H) Funnel plot for LDL cholesterol (ebi‐a‐GCST90018961) with meniscal injuries. (I) Funnel plot for LDL cholesterol (ebia‐GCST90092814) with meniscal injuries. (J) Funnel plot for apolipoprotein A1 levels with meniscal injuries. (K) Funnel plot for apolipoprotein B levels with meniscal injuries. (L) Funnel plot for fasting glucose with meniscal injuries. (M) Funnel plot for calcium levels with meniscal injuries.

**Supporting Information 6** Figure S6: Leave‐one‐out plots to visualize the causal effects of circulating metabolic factors on meniscal injuries (Part 1). (A) Leave‐one‐out analysis for uric acid with meniscal injuries. (B) Leave‐one‐out analysis for bone mineral density with meniscal injuries. (C) Leave‐one‐out analysis for serum 25‐hydroxyvitamin D levels with meniscal injuries. (D) Leave‐one‐out analysis for TC with meniscal injuries. (E) Leave‐one‐out analysis for triglycerides (ebi‐a‐GCST90018975) with meniscal injuries. (F) Leave‐one‐out analysis for triglycerides (ebi‐aGCST90092992) with meniscal injuries.

**Supporting Information 7** Figure S7: Leave‐one‐out plots to visualize the causal effects of circulating metabolic factors on meniscal injuries (Part 2). (A) Leave‐one‐out analysis for HDL cholesterol with meniscal injuries. (B) Leave‐one‐out analysis for LDL cholesterol (ebi‐a‐GCST90018961) with meniscal injuries. (C) Leave‐one‐out analysis for LDL cholesterol (ebi‐a‐GCST90092814) with meniscal injuries. (D) Leave‐one‐out analysis for apolipoprotein A1 levels with meniscal injuries. (E) Leave‐one‐out analysis for apolipoprotein B levels with meniscal injuries. (F) Leave‐one‐out analysis for fasting glucose with meniscal injuries. (G) Leave‐one‐out analysis for calcium levels with meniscal injuries.

**Supporting Information 8** Table S1: GWAS database of obesity‐related indicators.

**Supporting Information 9** Table S2: GWAS database of circulating metabolic factors.

**Supporting Information 10** Table S3: Instrumental variable screening of obesity‐related indicators on meniscal injuries and *F* test of instrumental variables.

**Supporting Information 11** Table S4: Instrumental variable screening of circulating metabolic factors on meniscal injuries and *F* test of instrumental variables.

**Supporting Information 12** Table S5: The causal effect estimation results of the three models of obesity‐related indicators on meniscal injuries.

**Supporting Information 13** Table S6: The causal effect estimation results of the three models of circulating metabolic factors on meniscal injuries.

**Supporting Information 14** Table S7: Estimation of MR causal effects of obesity‐related indicators on meniscal injuries (IVW fixed‐effects model).

**Supporting Information 15** Table S8: Estimation of MR causal effects of obesity‐related indicators on meniscal injuries (IVW random‐effects model).

**Supporting Information 16** Table S9: The Steiger directivity test of Mendelian randomized analysis of obesity‐related indicators on meniscal injuries.

**Supporting Information 17** Table S10: Estimation of MR causal effects of circulating metabolic factors on meniscal injuries (IVW fixed‐effects model).

**Supporting Information 18** Table S11: The Steiger directivity test of Mendelian randomized analysis of circulating metabolic indicators on meniscal injuries.

**Supporting Information 19** Table S12: MR analysis heterogeneity test of obesity‐related indicators for meniscal injuries.

**Supporting Information 20** Table S13: Pleiotropy test of MR analysis of obesity‐related indicators for meniscal injuries.

**Supporting Information 21** Table S14: MR analysis heterogeneity test of circulating metabolic indicators for meniscal injuries.

**Supporting Information 22** Table S15: Pleiotropy test of MR analysis of circulating metabolic factors for meniscal injuries.

**Supporting Information 23** Table S16: Pleiotropy test of MR of obesity‐related indicators on circulating metabolic factors.

**Supporting Information 24** Table S17: Heterogeneity test of MR of obesity‐related indicators for circulating metabolic indicators.

**Supporting Information 25** Table S18: The Steiger directivity test of MR of obesity‐related indicators on circulating metabolic factors.

**Supporting Information 26** Table S19: Instrumental variable screening of obesity‐related indicators on circulating metabolic factors and *F* test of instrumental variables.

**Supporting Information 27** Table S20: The causal effect estimation results of the three models of obesity‐related indicators on circulating metabolic factors.

**Supporting Information 28** Table S21: Estimation of MR causal effects of obesity‐related indicators on circulating metabolic factors (IVW fixed‐effects model).

**Supporting Information 29** Table S22: Estimation of MR causal effects of obesity‐related indicators on circulating metabolic factors (IVW random‐effects model).

**Supporting Information 30** Table S23: Sobel test of circulating metabolic indicators mediating effects of obesity‐related indicators on meniscal injuries.

**Supporting Information 31** Table S24: Effect of circulating metabolic indicators mediating obesity‐related indicators on meniscal injuries through MR.

## Data Availability

The datasets used and/or generated during the current study are presented in this published article.
