# Supplementary figures and images for: Circulating Metabolic Factors Mediating the Effect of Obesity‐Related Indicators on Meniscal Injuries: A Mendelian Randomization Study
Source: Int J Genomics. 2026 Feb 23;2026:8056288. doi: 10.1155/ijog/8056288 (PMC12929031; doi:10.1155/ijog/8056288)

A

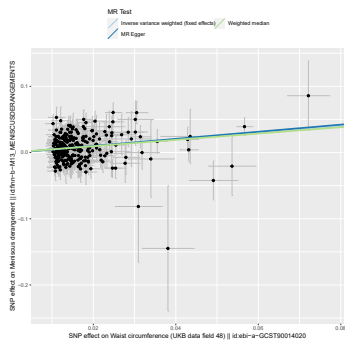

B

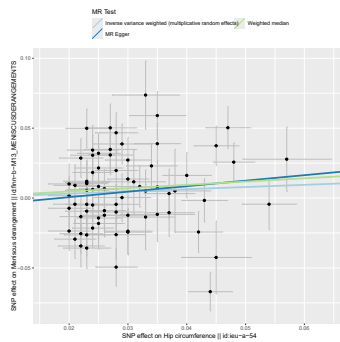

C

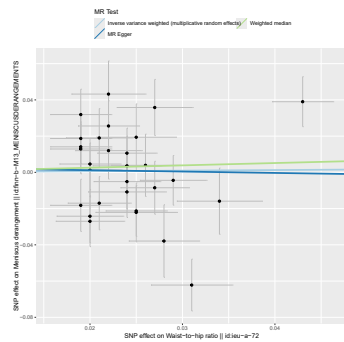

D

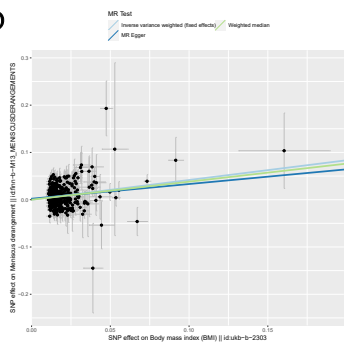

E

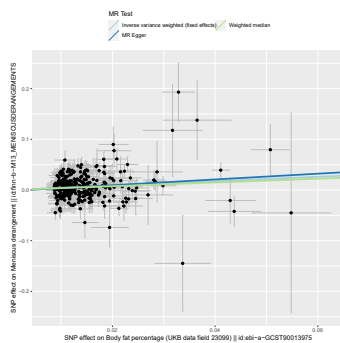

F

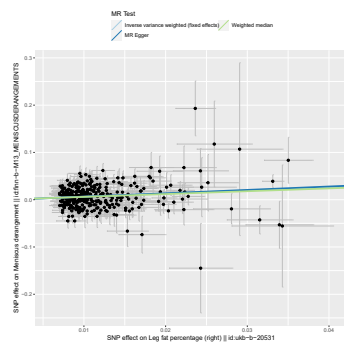

G

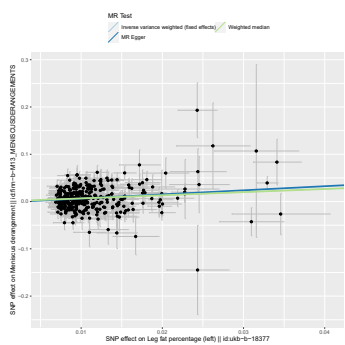

Supplement: Supplementary file 1 — Supporting Information 1 Figure S1: Scatter plot of correlation between obesity‐related indicators and meniscal injuries. (A) Scatter plot for waist circumference with meniscal injuries. (B) Scatter plot for hip circumference with meniscal injuries. (C) Scatter plot for waist‐to‐hip ratio with meniscal injuries. (D) Scatter plot for BMI with meniscal injuries. (E) Scatter plot for body fat percentage with meniscal injuries. (F) Scatter plot for leg fat percentage (right) with meniscal injuries. (G) Scatter plot for leg fat percentage (left) with meniscal injuries. [file IJOG-2026-8056288-s029.pdf]

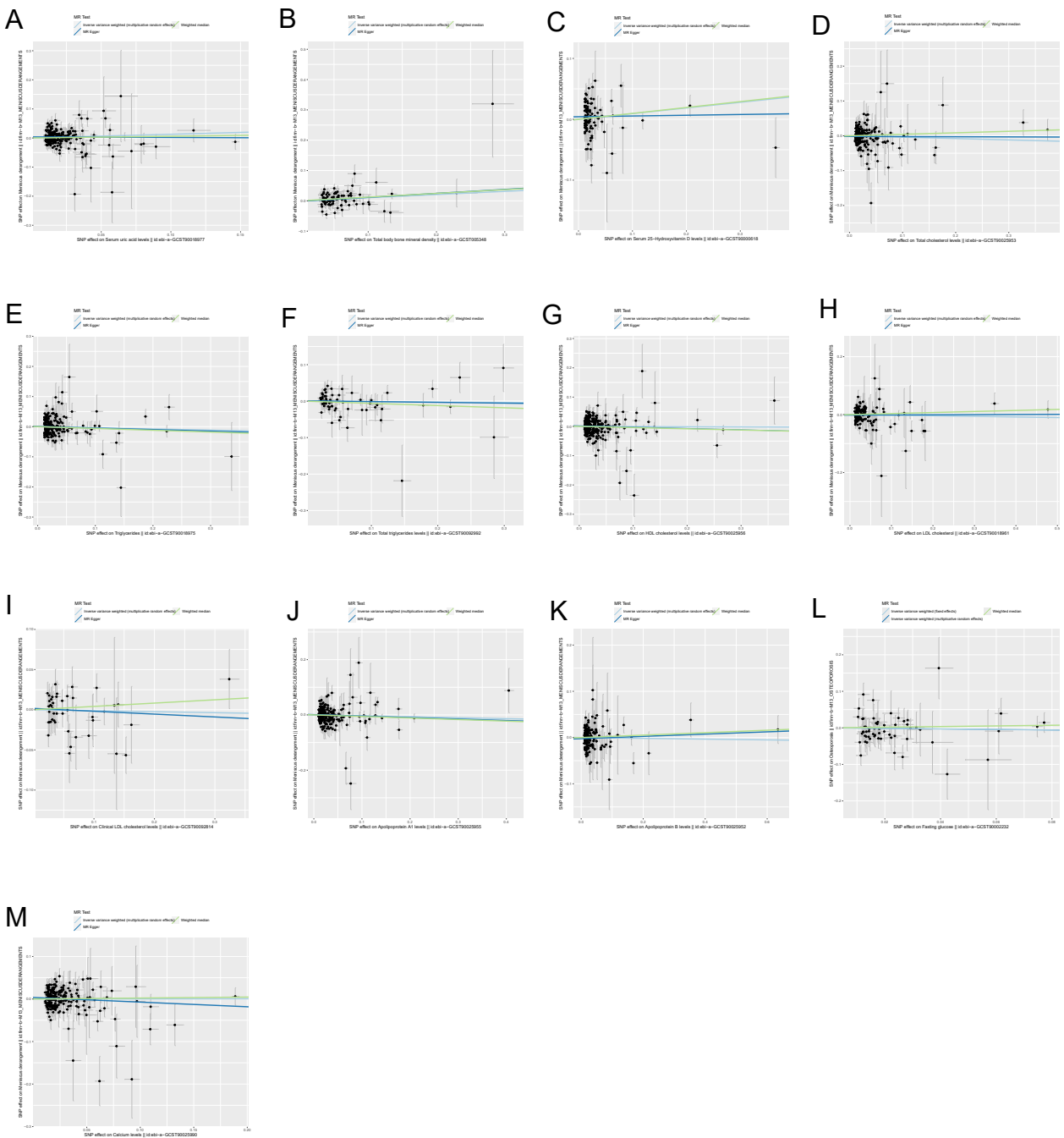

Supplement: Supplementary file 2 — Supporting Information 2 Figure S2: Scatter plot of correlation between circulating metabolic factors and meniscal injuries. (A) Scatter plot for uric acid with meniscal injuries. (B) Scatter plot for bone mineral density with meniscal injuries. (C) Scatter plot for serum 25‐hydroxyvitamin D levels with meniscal injuries. (D) Scatter plot for TC with meniscal injuries. (E) Scatter plot for triglycerides (ebi‐aGCST90018975) with meniscal injuries. (F) Scatter plot for triglycerides (ebi‐aGCST90092992) with meniscal injuries. (G) Scatter plot for HDL cholesterol with meniscal injuries. (H) Scatter plot for LDL cholesterol (ebi‐a‐GCST90018961) with meniscal injuries. (I) Scatter plot for LDL cholesterol (ebia‐GCST90092814) with meniscal injuries. (J) Scatter plot for apolipoprotein A1 levels with meniscal injuries. (K) Scatter plot for apolipoprotein B levels with meniscal injuries. (L) Scatter plot for fasting glucose with meniscal injuries. (M) Scatter plot for calcium levels with meniscal injuries. [file IJOG-2026-8056288-s028.pdf]

**A**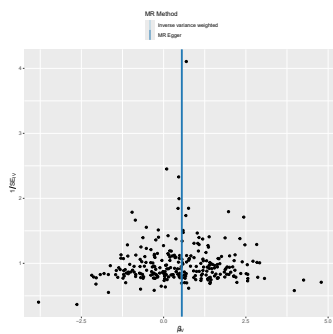**B**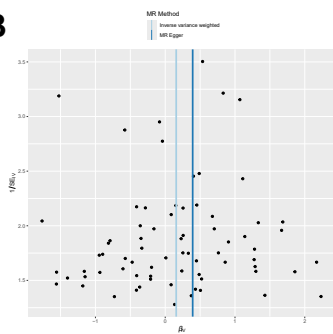**C**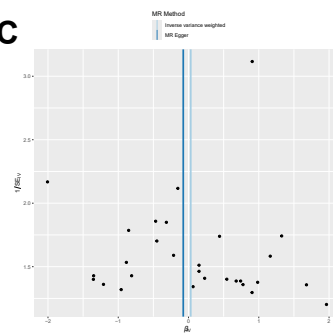**D**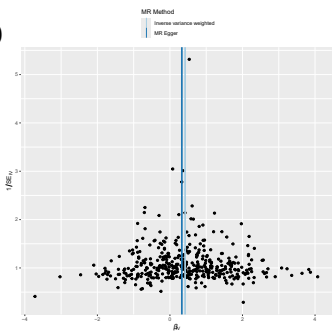**E**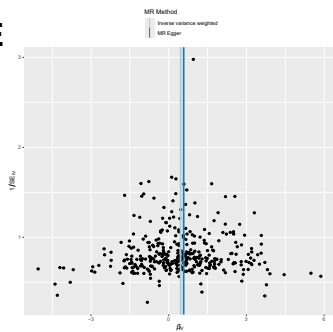**F**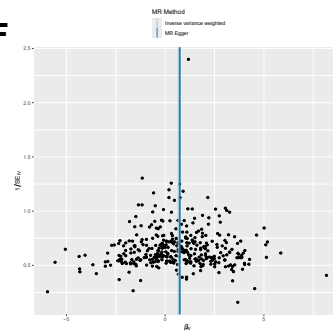**G**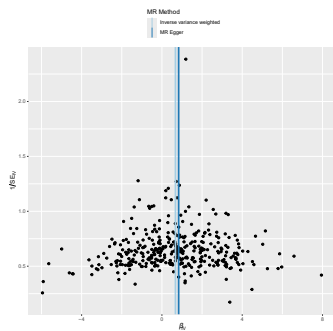

Supplement: Supplementary file 3 — Supporting Information 3 Figure S3: Funnel plot of heterogeneity test for MR of obesity‐related indicators on meniscal injuries. (A) Funnel plot for waist circumference with meniscal injuries. (B) Funnel plot for hip circumference with meniscal injuries. (C) Funnel plot for waist‐to‐hip ratio with meniscal injuries. (D) Funnel plot for BMI with meniscal injuries. (E) Funnel plot for body fat percentage with meniscal injuries. (F) Funnel plot for leg fat percentage (right) with meniscal injuries. (G) Funnel plot for leg fat percentage (left) with meniscal injuries. [file IJOG-2026-8056288-s030.pdf]

**A**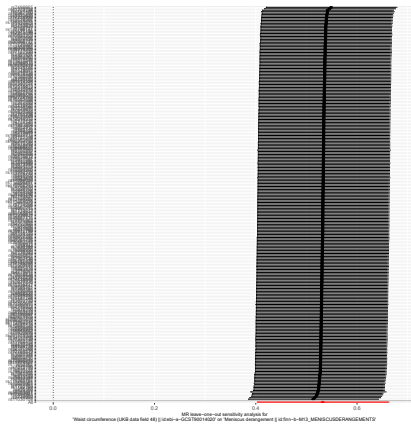**B**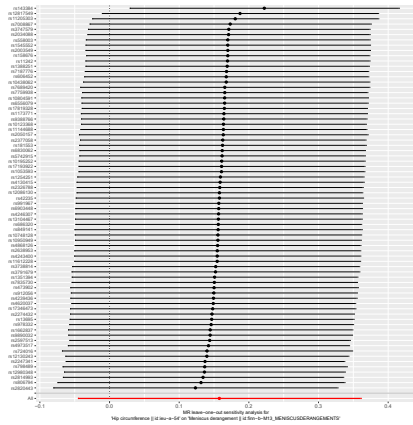**C**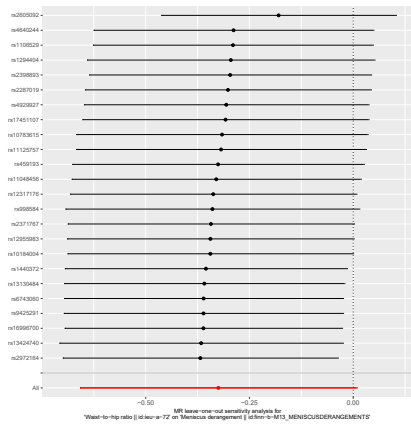**D**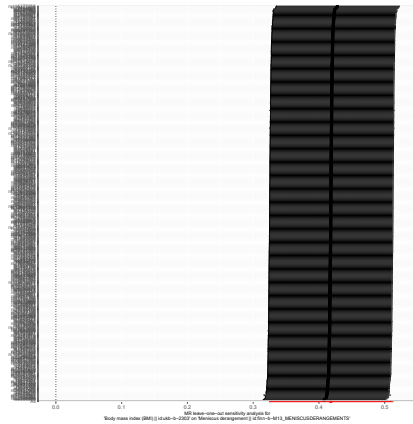**E**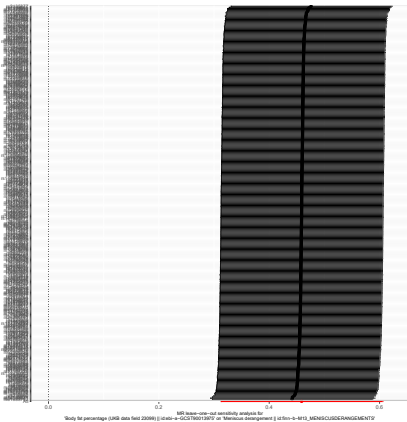**F**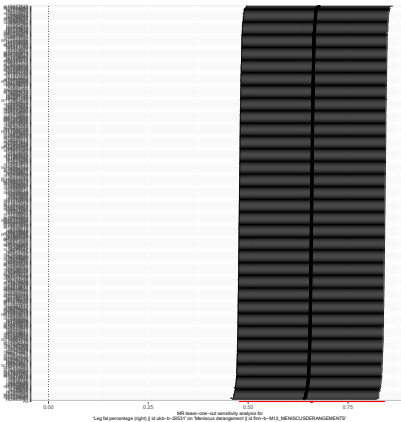**G**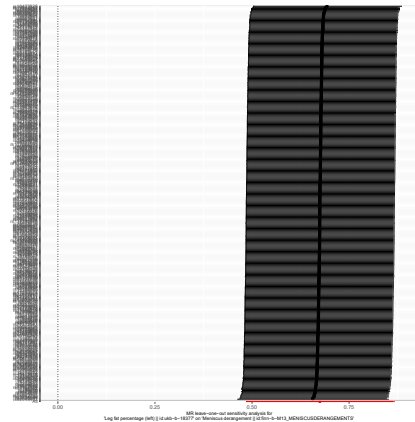

Supplement: Supplementary file 4 — Supporting Information 4 Figure S4: Leave‐one‐out plots to visualize the causal effects of obesity‐related indicators on meniscal injuries. (A) Leave‐one‐out analysis for waist circumference with meniscal injuries. (B) Leave‐one‐out analysis for hip circumference with meniscal injuries. (C) Leave‐one‐out analysis for waist‐to‐hip ratio with meniscal injuries. (D) Leave‐one‐out analysis for BMI with meniscal injuries. (E) Leave‐one‐out analysis for body fat percentage with meniscal injuries. (F) Leave‐one‐out analysis for leg fat percentage (right) with meniscal injuries. (G) Leave‐one‐out analysis for leg fat percentage (left) with meniscal injuries. [file IJOG-2026-8056288-s031.pdf]

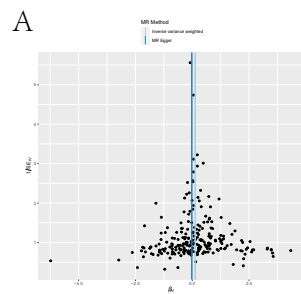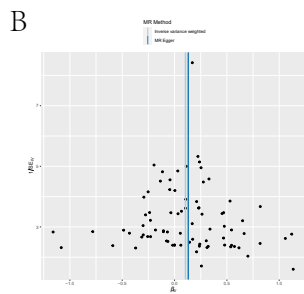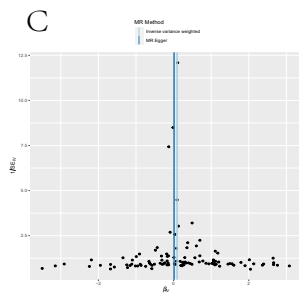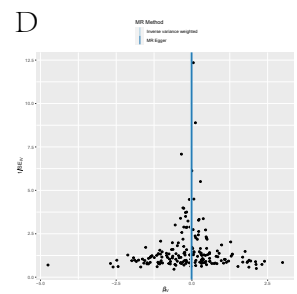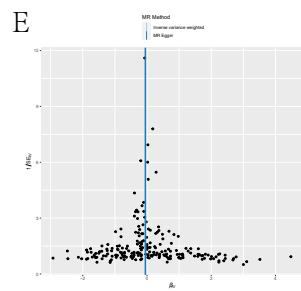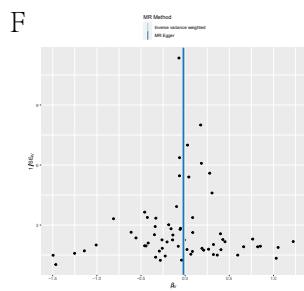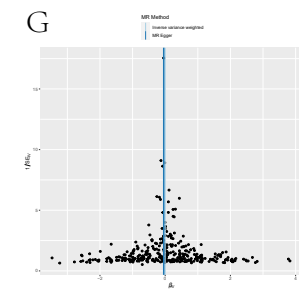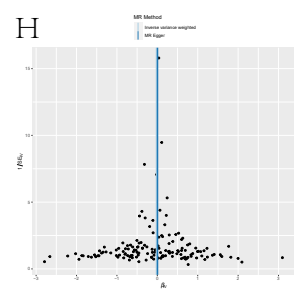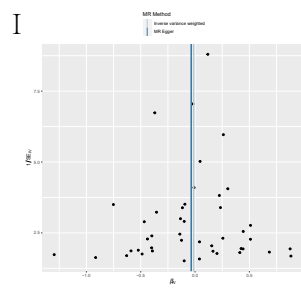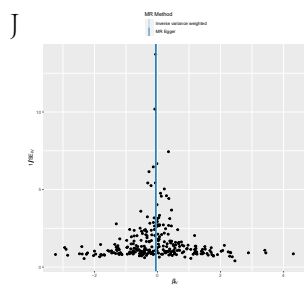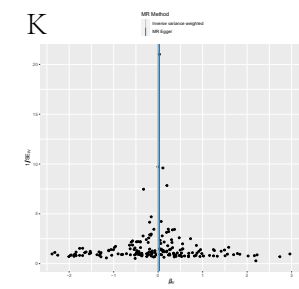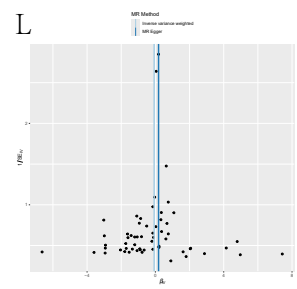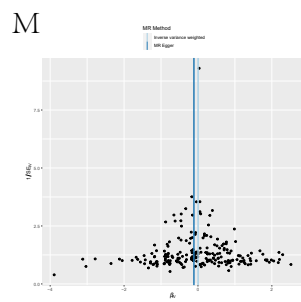

Supplement: Supplementary file 5 — Supporting Information 5 Figure S5: Funnel plot of heterogeneity test for MR of circulating metabolic factors on meniscal injuries. (A) Funnel plot for uric acid with meniscal injuries. (B) Funnel plot for bone mineral density with meniscal injuries. (C) Funnel plot for serum 25‐hydroxyvitamin D levels with meniscal injuries. (D) Funnel plot for TC with meniscal injuries. (E) Funnel plot for triglycerides (ebi‐aGCST90018975) with meniscal injuries. (F) Funnel plot for triglycerides (ebi‐aGCST90092992) with meniscal injuries. (G) Funnel plot for HDL cholesterol with meniscal injuries. (H) Funnel plot for LDL cholesterol (ebi‐a‐GCST90018961) with meniscal injuries. (I) Funnel plot for LDL cholesterol (ebia‐GCST90092814) with meniscal injuries. (J) Funnel plot for apolipoprotein A1 levels with meniscal injuries. (K) Funnel plot for apolipoprotein B levels with meniscal injuries. (L) Funnel plot for fasting glucose with meniscal injuries. (M) Funnel plot for calcium levels with meniscal injuries. [file IJOG-2026-8056288-s001.pdf]

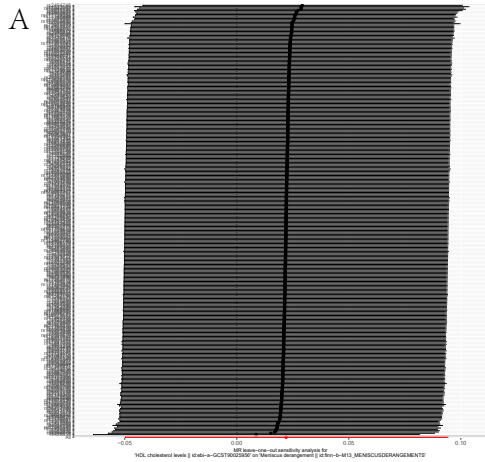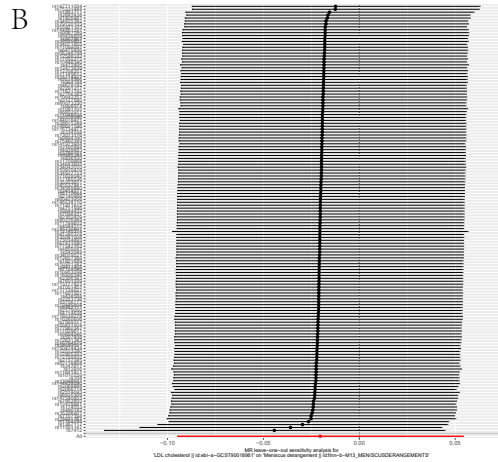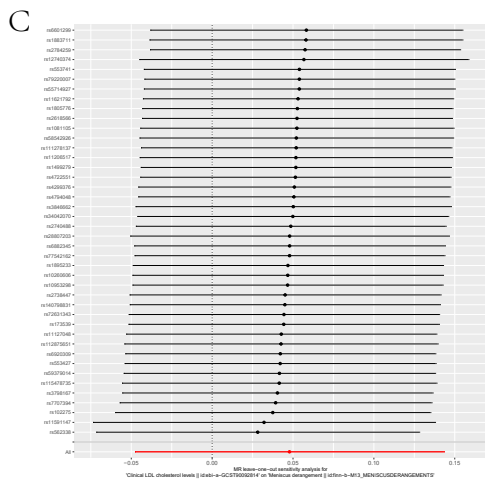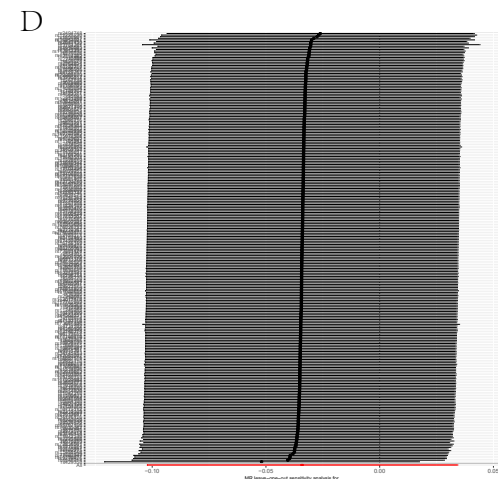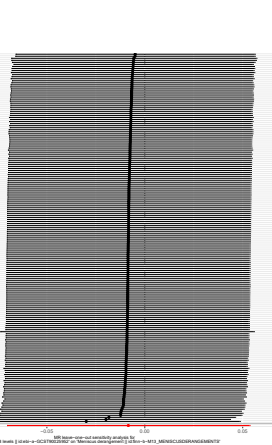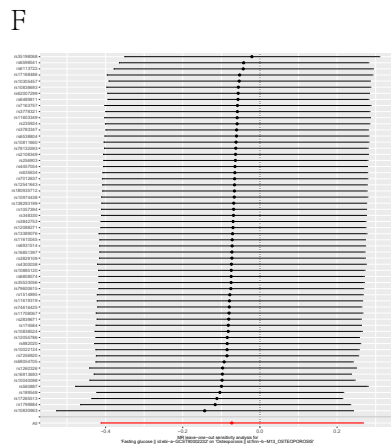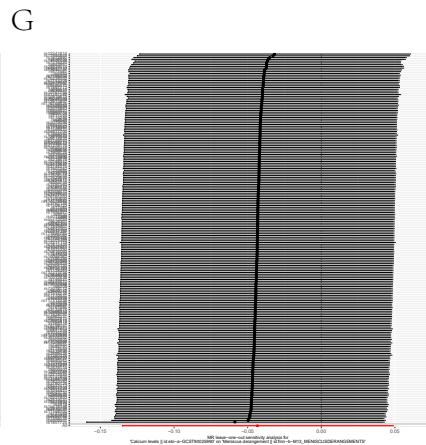

Supplement: Supplementary file 7 — Supporting Information 7 Figure S7: Leave‐one‐out plots to visualize the causal effects of circulating metabolic factors on meniscal injuries (Part 2). (A) Leave‐one‐out analysis for HDL cholesterol with meniscal injuries. (B) Leave‐one‐out analysis for LDL cholesterol (ebi‐a‐GCST90018961) with meniscal injuries. (C) Leave‐one‐out analysis for LDL cholesterol (ebi‐a‐GCST90092814) with meniscal injuries. (D) Leave‐one‐out analysis for apolipoprotein A1 levels with meniscal injuries. (E) Leave‐one‐out analysis for apolipoprotein B levels with meniscal injuries. (F) Leave‐one‐out analysis for fasting glucose with meniscal injuries. (G) Leave‐one‐out analysis for calcium levels with meniscal injuries. [file IJOG-2026-8056288-s003.pdf]
