# Supplementary material for: Circulating Metabolic Factors Mediating the Effect of Obesity‐Related Indicators on Meniscal Injuries: A Mendelian Randomization Study
Source: Int J Genomics. 2026 Feb 23;2026:8056288. doi: 10.1155/ijog/8056288 (PMC12929031; doi:10.1155/ijog/8056288)
Supplement: Supplementary file 6 — Supporting Information 6 Figure S6: Leave‐one‐out plots to visualize the causal effects of circulating metabolic factors on meniscal injuries (Part 1). (A) Leave‐one‐out analysis for uric acid with meniscal injuries. (B) Leave‐one‐out analysis for bone mineral density with meniscal injuries. (C) Leave‐one‐out analysis for serum 25‐hydroxyvitamin D levels with meniscal injuries. (D) Leave‐one‐out analysis for TC with meniscal injuries. (E) Leave‐one‐out analysis for triglycerides (ebi‐a‐GCST90018975) with meniscal injuries. (F) Leave‐one‐out analysis for triglycerides (ebi‐aGCST90092992) with meniscal injuries. [file IJOG-2026-8056288-s002.pdf]

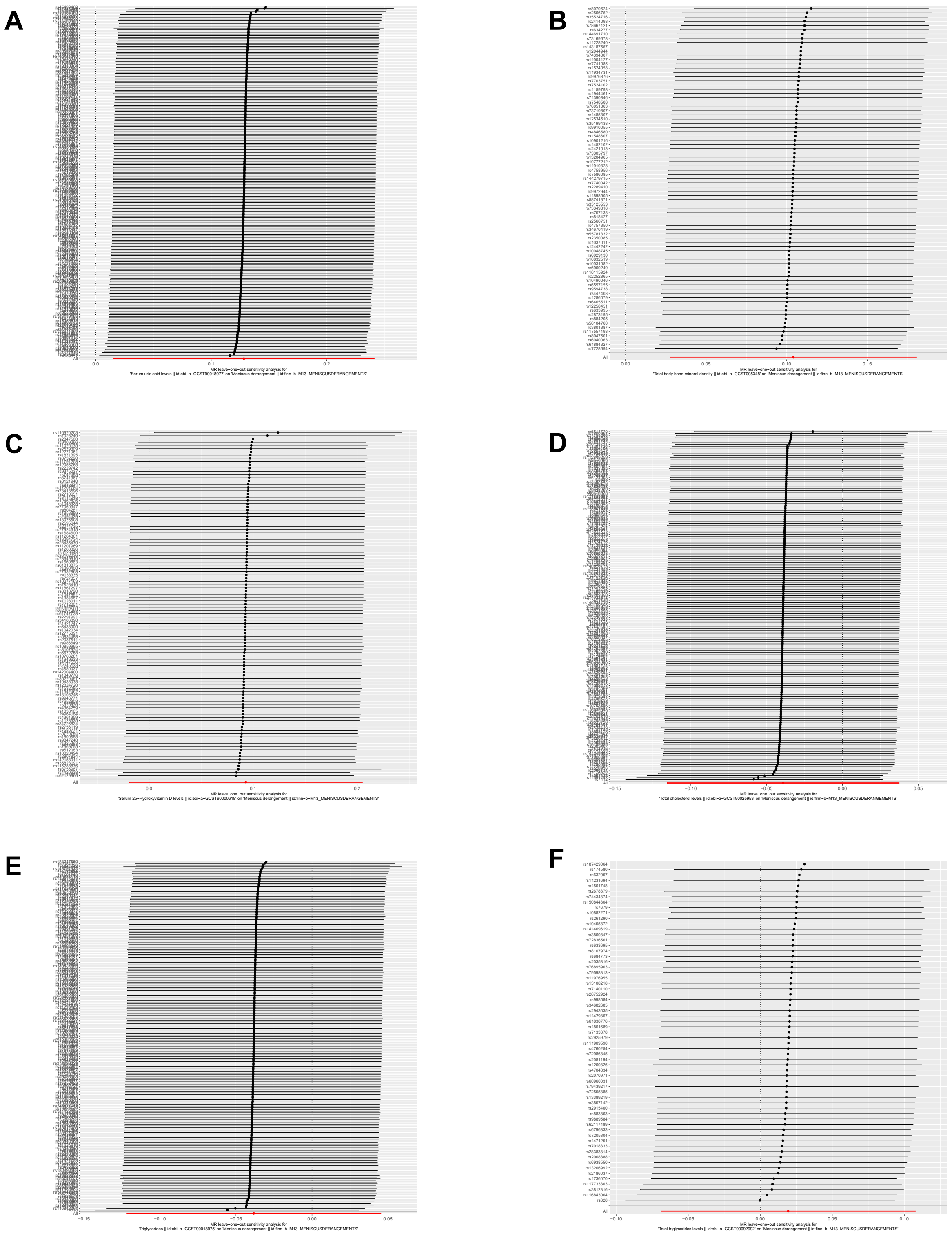
A
All
0.0
0.1
0.2
MR leave-one-out sensitivity analysis for
'Serum uric acid levels || id:ebi-a-GCST90018977' on 'Meniscus derangement || id:finn-b-M13_MENISCUSDERANGEMENTS'
B
All
0.00
0.05
0.10
0.15
MR leave-one-out sensitivity analysis for
'Total body bone mineral density || id:ebi-a-GCST005348' on 'Meniscus derangement || id:finn-b-M13_MENISCUSDERANGEMENTS'
C
All
0.0
0.1
0.2
MR leave-one-out sensitivity analysis for
'Serum 25-Hydroxyvitamin D levels || id:ebi-a-GCST90000618' on 'Meniscus derangement || id:finn-b-M13_MENISCUSDERANGEMENTS'
D
All
-0.15
-0.10
-0.05
0.00
0.05
MR leave-one-out sensitivity analysis for
'Total cholesterol levels || id:ebi-a-GCST90025953' on 'Meniscus derangement || id:finn-b-M13_MENISCUSDERANGEMENTS'
E
All
-0.15
-0.10
-0.05
0.00
0.05
MR leave-one-out sensitivity analysis for
'Triglycerides || id:ebi-a-GCST90018975' on 'Meniscus derangement || id:finn-b-M13_MENISCUSDERANGEMENTS'
F
All
-0.10
-0.05
0.00
0.05
0.10
MR leave-one-out sensitivity analysis for
'Total triglycerides levels || id:ebi-a-GCST90092992' on 'Meniscus derangement || id:finn-b-M13_MENISCUSDERANGEMENTS'
