# Supplementary material for: Circulating Metabolic Factors Mediating the Effect of Obesity‐Related Indicators on Meniscal Injuries: A Mendelian Randomization Study
Source: Int J Genomics. 2026 Feb 23;2026:8056288. doi: 10.1155/ijog/8056288 (PMC12929031; doi:10.1155/ijog/8056288)
Supplement: Supplementary file 10 — Supporting Information 10 Table S3: Instrumental variable screening of obesity‐related indicators on meniscal injuries and F test of instrumental variables. [file IJOG-2026-8056288-s006.docx]

**Table S3.** **Instrumental variables screening of obesity-related indicators on meniscal injuries and F test of instrumental variables**

| **Exposure** | **Number of SNPs** | **Median of F** | **Fmin** | **Fmax** |
| --- | --- | --- | --- | --- |
| **Waist circumference\|\|ebi-a-GCST90014020** | 285 | 41.13103 | 14.4537 | 643.9648 |
| **hip circumference\|\|ieu-a-54** | 73 | 73.88567 | 34.2082 | 253.4627 |
| **waist-to-hip ratio\|\|ieu-a-72** | 24 | 53.43616 | 30.6532 | 205.4861 |
| **BMI\|\|ukb-b-2303** | 403 | 58.12147 | 27.0151 | 1182.0375 |
| **Body fat percentage\|\|ebi-a-GCST90013975** | 344 | 28.97756 | 13.4225 | 333.7484 |
| **Leg fat percentage(right)\|\|ukb-b-20531** | 351 | 20.47798 | 11.0859 | 240.2774 |
| **Leg fat percentage(left)\|\|ukb-b-18377** | 350 | 20.11126 | 10.5748 | 237.3897 |

SNPs：Single Nucleotide Polymorphisms；F：F statistics.
