# Supplementary material for: Circulating Metabolic Factors Mediating the Effect of Obesity‐Related Indicators on Meniscal Injuries: A Mendelian Randomization Study
Source: Int J Genomics. 2026 Feb 23;2026:8056288. doi: 10.1155/ijog/8056288 (PMC12929031; doi:10.1155/ijog/8056288)
Supplement: Supplementary file 11 — Supporting Information 11 Table S4: Instrumental variable screening of circulating metabolic factors on meniscal injuries and F test of instrumental variables. [file IJOG-2026-8056288-s007.docx]

**Table S4. Instrumental variables screening of circulating metabolic factors on meniscal injuries and F test of instrumental variables**

| **Exposure** | **Number of SNPs** | **Median of F** | **Fmin** | **Fmax** |
| --- | --- | --- | --- | --- |
| **uric acid \|\|ebi-a-GCST90018977** | 231 | 60.5635 | 16.15274036 | 2024.7471 |
| **Bone mineral density\|\|ebi-a-GCST005348** | 81 | 63.2516 | 26.5407648 | 407.4437 |
| **Serum 25-Hydroxyvitamin D levels\|\|ebi-a-GCST90000618** | 107 | 214.5007 | 30.4597 | 8917.7479 |
| **TC\|\|ebi-a-GCST90025953** | 190 | 190.6489 | 22.30078033 | 9136.5137 |
| **Triglycerides\|\|ebi-a-GCST90018975** | 212 | 133.8831 | 21.23654119 | 4899.1563 |
| **Triglycerides\|\|ebi-a-GCST90092992** | 63 | 127.9798 | 27.8132 | 1284.8353 |
| **HDL cholesterol\|\|ebi-a-GCST90025956** | 329 | 182.0271 | 132.8706 | 3188.4174 |
| **LDL cholesterol\|\|ebi-a-GCST90018961** | 147 | 201.6608 | 23.29248 | 10934.0308 |
| **LDL cholesterol\|\| ebi-a-GCST90092814** | 42 | 98.5416 | 29.66434 | 545.3215 |
| **Apolipoprotein A1 levels\|\|\|ebi-a-GCST90025955** | 269 | 175.9065 | 19.51514 | 7733.9488 |
| **Apolipoprotein B levels\|\|ebi-a-GCST90025952** | 182 | 310.5486 | 18.60094846 | 27157.7486 |
| **Fasting glucose\|\|ebi-a-GCST90002232** | 59 | 44.0644 | 10.55875441 | 489.5099 |
| **Calcium levels\|\| ebi-a-GCST90025990** | 210 | 92.7962 | 25.0707731 | 3270.8069 |

SNPs：Single Nucleotide Polymorphisms；F：F statistics.
