# Supplementary material for: Circulating Metabolic Factors Mediating the Effect of Obesity‐Related Indicators on Meniscal Injuries: A Mendelian Randomization Study
Source: Int J Genomics. 2026 Feb 23;2026:8056288. doi: 10.1155/ijog/8056288 (PMC12929031; doi:10.1155/ijog/8056288)
Supplement: Supplementary file 14 — Supporting Information 14 Table S7: Estimation of MR causal effects of obesity‐related indicators on meniscal injuries (IVW fixed‐effects model). [file IJOG-2026-8056288-s010.docx]

**Table S7**. Estimation of MR causal effects of obesity-related indicators on meniscal injuries (IVW random-effects model).

| **Exposure** | **ID** | **Number of SNPs** | **β** | **Standard error** | **OR (95%CI)** | ***p*-value** |
| --- | --- | --- | --- | --- | --- | --- |
| **Waist circumference** | ebi-a-GCST90014020 | 285 | 0.4906052 | 0.0571545 | 1.6333(1.4602,1.8269) | 0.0000 |
| **BMI** | ukb-b-2303 | 403 | 0.4171038 | 0.0431168 | 1.5175(1.3945,1.6513) | 0.0000 |
| **Body fat percentage** | ebi-a-GCST90013975 | 344 | 0.4350009 | 0.0620033 | 1.5449(1.3682,1.7446) | 0.0000 |
| **Leg fat percentage(right)** | ukb-b-20531 | 351 | 0.6470418 | 0.0776539 | 1.9099(1.6402,2.2239) | 0.0000 |
| **Leg fat percentage(left)** | ukb-b-18377 | 350 | 0.6643341 | 0.0787492 | 1.9432(1.6652,2.2675) | 0.0000 |

SNP, single-nucleotide polymorphism; OR, odds ratio; CI, confidence interval.
