# Supplementary material for: Circulating Metabolic Factors Mediating the Effect of Obesity‐Related Indicators on Meniscal Injuries: A Mendelian Randomization Study
Source: Int J Genomics. 2026 Feb 23;2026:8056288. doi: 10.1155/ijog/8056288 (PMC12929031; doi:10.1155/ijog/8056288)
Supplement: Supplementary file 15 — Supporting Information 15 Table S8: Estimation of MR causal effects of obesity‐related indicators on meniscal injuries (IVW random‐effects model). [file IJOG-2026-8056288-s011.docx]

**Table S8**. Estimation of MR causal effects of obesity-related indicators on meniscal injuries (IVW random-effects model).

| **Exposure** | **ID** | **Number of SNPs** | **β** | **Standard error** | **OR (95%CI)** | ***p*-value** |
| --- | --- | --- | --- | --- | --- | --- |
| **hip circumference** | ieu-a-54 | 73 | 0.1576442 | 0.1035879 | 1.1707(0.9556,1.4343) | 0.1280 |
| **waist-to-hip ratio** | ieu-a-72 | 24 | 0.0317549 | 0.1832066 | 1.0322(0.7208,1.4782) | 0.8624 |

SNP, single-nucleotide polymorphism; OR, odds ratio; CI, confidence interval.
