# Supplementary material for: Circulating Metabolic Factors Mediating the Effect of Obesity‐Related Indicators on Meniscal Injuries: A Mendelian Randomization Study
Source: Int J Genomics. 2026 Feb 23;2026:8056288. doi: 10.1155/ijog/8056288 (PMC12929031; doi:10.1155/ijog/8056288)
Supplement: Supplementary file 16 — Supporting Information 16 Table S9: The Steiger directivity test of Mendelian randomized analysis of obesity‐related indicators on meniscal injuries. [file IJOG-2026-8056288-s012.docx]

**Table S9. The Steiger directivity test of Mendelian randomized analysis of obesity-related indicators on meniscal injuries**

| **Exposure** | **Steiger p value** | **SNP r2**  **exposure** | **SNP r2 outcome** | **Correct causal direction** |
| --- | --- | --- | --- | --- |
| **Waist circumference\|\|ebi-a-GCST90014020** | 0 | 0.040741 | 0.00633 | TRUE |
| **hip circumference\|\|ieu-a-54** | 1.73E-195 | 0.0187472 | 0.00312 | TRUE |
| **waist-to-hip ratio\|\|ieu-a-72** | 2.11E-61 | 0.0059627 | 0.00103 | TRUE |
| **BMI\|\|ukb-b-2303** | 0 | 0.0558611 | 0.00867 | TRUE |
| **Body fat percentage\|\|ebi-a-GCST90013975** | 0 | 0.04886 | 0.00817 | TRUE |
| **Leg fat percentage(right)\|\|ukb-b-20531** | 0 | 0.042493 | 0.00844 | TRUE |
| **Leg fat percentage(left)\|\|ukb-b-18377** | 0 | 0.042731 | 0.00891 | TRUE |

SNP: single nucleotide polymorphism
