# Supplementary material for: Circulating Metabolic Factors Mediating the Effect of Obesity‐Related Indicators on Meniscal Injuries: A Mendelian Randomization Study
Source: Int J Genomics. 2026 Feb 23;2026:8056288. doi: 10.1155/ijog/8056288 (PMC12929031; doi:10.1155/ijog/8056288)
Supplement: Supplementary file 17 — Supporting Information 17 Table S10: Estimation of MR causal effects of circulating metabolic factors on meniscal injuries (IVW fixed‐effects model). [file IJOG-2026-8056288-s013.docx]

**Table S10**. Estimation of MR causal effects of circulating metabolic factors on meniscal injuries (IVW fixed-effects model).

| **Exposure** | **ID** | **Number of SNPs** | **β** | **Standard error** | **OR (95%CI)** | ***p*-value** |
| --- | --- | --- | --- | --- | --- | --- |
| **uric acid** | ebi-a-GCST90018977 | 231 | 0.128339 | 0.050726 | 1.1369(1.0293,1.2557) | 0.011 |
| **Bone mineral density** | ebi-a-GCST005348 | 81 | 0.104203 | 0.032787 | 1.1098(1.0407,1.1834) | 0.001 |
| **Serum 25-Hydroxyvitamin D levels** | ebi-a-GCST90000618 | 107 | 0.092936 | 0.047247 | 1.0974(1.0003,1.2039) | 0.185 |
| **TC** | ebi-a-GCST90025953 | 190 | -0.0392 | 0.034702 | 0.9616(0.8983,1.0292) | 0.258 |
| **Triglycerides** | ebi-a-GCST90018975 | 212 | -0.0383 | 0.034777 | 0.9624(0.8990,1.0303) | 0.271 |
| **Triglycerides** | ebi-a-GCST90092992 | 63 | -0.00944 | 0.037401 | 0.9905(0.9205,1.0659) | 0.801 |
| **HDL cholesterol** | ebi-a-GCST90025956 | 329 | -0.00544 | 0.02527 | 0.9945(0.9465,1.0451) | 0.829 |
| **LDL cholesterol** | ebi-a-GCST90018961 | 147 | -0.02045 | 0.034461 | 0.9797(0.9158,1.0482) | 0.553 |
| **LDL cholesterol** | ebi-a-GCST90092814 | 42 | -0.01225 | 0.045705 | 0.9878(0.9031,1.0803) | 0.789 |
| **Apolipoprotein A1 levels** | ebi-a-GCST90025955 | 269 | -0.0341 | 0.02831 | 0.9664(0.9143,1.0216) | 0.228 |
| **Apolipoprotein B levels** | ebi-a-GCST90025952 | 182 | -0.0084 | 0.029175 | 0.9916(0.9365,1.0499) | 0.773 |
| **Fasting glucose** | ebi-a-GCST90002232 | 59 | -0.0728 | 0.162140 | 0.9297(0.6766, 1.2775) | 0.653 |
| **Calcium levels** | ebi-a-GCST90025990 | 210 | 0.00306 | 0.043049 | 1.0031(0.9219,1.0914) | 0.943337 |

SNP, single-nucleotide polymorphism; OR, odds ratio; CI, confidence interval.
