# Supplementary material for: Circulating Metabolic Factors Mediating the Effect of Obesity‐Related Indicators on Meniscal Injuries: A Mendelian Randomization Study
Source: Int J Genomics. 2026 Feb 23;2026:8056288. doi: 10.1155/ijog/8056288 (PMC12929031; doi:10.1155/ijog/8056288)
Supplement: Supplementary file 18 — Supporting Information 18 Table S11: The Steiger directivity test of Mendelian randomized analysis of circulating metabolic indicators on meniscal injuries. [file IJOG-2026-8056288-s014.docx]

**Table S11. The Steiger directivity test of Mendelian randomized analysis of circulating metabolic indicators on meniscal injuries**

| **Exposure** | **Steiger p value** | **SNP r2 exposure** | **SNP r2 outcome** | **Correct causal direction** |
| --- | --- | --- | --- | --- |
| **uric acid \|\|ebi-a-GCST90018977** | 0 | 0.060101 | 0.004339 | TRUE |
| **Bone mineral density\|\|ebi-a-GCST005348** | 0 | 0.094941 | 0.001763 | TRUE |
| **Serum 25-Hydroxyvitamin D levels\|\|ebi-a-GCST90000618** | 0 | 0.021514 | 0.002267 | TRUE |
| **TC\|\|ebi-a-GCST90025953** | 0 | 0.062209 | 0.003423 | TRUE |
| **Triglycerides\|\|ebi-a-GCST90018975** | 0 | 0.074689 | 0.004535 | TRUE |
| **Triglycerides\|\|ebi-a-GCST90092992** | 0 | 0.074121 | 0.001171 | TRUE |
| **HDL cholesterol\|\|ebi-a-GCST90025956** | 0 | 0.124668 | 0.007226 | TRUE |
| **LDL cholesterol\|\|ebi-a-GCST90018961** | 0 | 0.056128 | 0.002532 | TRUE |
| **LDL cholesterol\|\|ebi-a-GCST90092814** | 0 | 0.04283 | 0.000741 | TRUE |
| **Apolipoprotein A1 levels\|\|\|ebi-a-GCST90025955** | 0 | 0.101612 | 0.005772 | TRUE |
| **Apolipoprotein B levels\|\|ebi-a-GCST90025952** | 0 | 0.060229 | 0.003012 | TRUE |
| **Fasting glucose \|\| id:ebi-a-GCST90002232** | 0 | 0.035982 | 0.000192 | TRUE |
| **Calcium levels\|\|ebi-a-GCST90025990** | 0 | 0.049699 | 0.003972 | TRUE |

SNP: single nucleotide polymorphism
