# Supplementary material for: Circulating Metabolic Factors Mediating the Effect of Obesity‐Related Indicators on Meniscal Injuries: A Mendelian Randomization Study
Source: Int J Genomics. 2026 Feb 23;2026:8056288. doi: 10.1155/ijog/8056288 (PMC12929031; doi:10.1155/ijog/8056288)
Supplement: Supplementary file 19 — Supporting Information 19 Table S12: MR analysis heterogeneity test of obesity‐related indicators for meniscal injuries. [file IJOG-2026-8056288-s015.docx]

**Table S12. MR analysis heterogeneity test of obesity-related indicators for meniscal injuries**

| **Exposure** | **Q** | **Q_df** | **Q_pval** | **I^2^（%）** |
| --- | --- | --- | --- | --- |
| **Waist circumference\|\|ebi-a-GCST90014020** | 365.0288 | 283 | 0.000713 | 22.47% |
| **hip circumference\|\|ieu-a-54** | 211.0666 | 71 | 8.00E-16 | 66.36% |
| **waist-to-hip ratio\|\|ieu-a-72** | 72.93566 | 27 | 4.24E-06 | 62.98% |
| **BMI\|\|ukb-b-2303** | 515.3721 | 401 | 9.53E-05 | 22.19% |
| **Body fat percentage\|\|ebi-a-GCST90013975** | 518.3606 | 342 | 2.22E-09 | 34.02% |
| **Leg fat percentage(right)\|\|ukb-b-20531** | 517.7081 | 349 | 1.07E-08 | 32.59% |
| **Leg fat percentage(left)\|\|ukb-b-18377** | 548.8237 | 348 | 3.36E-11 | 36.59% |

Q: Cochran Q test；Q_df: degrees of freedom of Q test; Q_pval: P valve of Q test
