# Supplementary material for: Circulating Metabolic Factors Mediating the Effect of Obesity‐Related Indicators on Meniscal Injuries: A Mendelian Randomization Study
Source: Int J Genomics. 2026 Feb 23;2026:8056288. doi: 10.1155/ijog/8056288 (PMC12929031; doi:10.1155/ijog/8056288)
Supplement: Supplementary file 20 — Supporting Information 20 Table S13: Pleiotropy test of MR analysis of obesity‐related indicators for meniscal injuries. [file IJOG-2026-8056288-s016.docx]

**Table S13. Pleiotropy test of MR analysis of obesity-related indicators for meniscal injuries**

| **Exposure** | **MR-Egger intercept** | **Standard error** | **pval** |
| --- | --- | --- | --- |
| **Waist circumference\|\|ebi-a-GCST90014020** | -0.00077 | 0.003015 | 0.798 |
| **hip circumference\|\|ieu-a-54** | -0.00724 | 0.011613 | 0.535 |
| **waist-to-hip ratio\|\|ieu-a-72** | 0.00268 | 0.020711 | 0.898 |
| **BMI\|\|ukb-b-2303** | 0.002096 | 0.002372 | 0.377 |
| **Body fat percentage\|\|ebi-a-GCST90013975** | -0.00191 | 0.003418 | 0.577 |
| **Leg fat percentage(right)\|\|ukb-b-20531** | -0.00082 | 0.003402 | 0.810 |
| **Leg fat percentage(left)\|\|ukb-b-18377** | -0.00229 | 0.003568 | 0.521 |
