# Supplementary material for: Circulating Metabolic Factors Mediating the Effect of Obesity‐Related Indicators on Meniscal Injuries: A Mendelian Randomization Study
Source: Int J Genomics. 2026 Feb 23;2026:8056288. doi: 10.1155/ijog/8056288 (PMC12929031; doi:10.1155/ijog/8056288)
Supplement: Supplementary file 21 — Supporting Information 21 Table S14: MR analysis heterogeneity test of circulating metabolic indicators for meniscal injuries. [file IJOG-2026-8056288-s017.docx]

**Table S14. MR analysis heterogeneity test of circulating metabolic indicators for meniscal injuries.**

| **Exposure** | **Q** | **Q_df** | **Q_pval** | **I^2^（%）** |
| --- | --- | --- | --- | --- |
| **uric acid \|\|ebi-a-GCST90018977** | 294.5405 | 229 | 0.002244 | 22.25% |
| **Bone mineral density\|\|ebi-a-GCST005348** | 113.4421 | 79 | 0.00673 | 30.36% |
| **Serum 25-Hydroxyvitamin D levels\|\|ebi-a-GCST90000618** | 150.9613 | 105 | 0.002224 | 30.45% |
| **TC\|\|ebi-a-GCST90025953** | 238.1858 | 188 | 0.007721 | 21.07% |
| **Triglycerides\|\|ebi-a-GCST90018975** | 316.9726 | 210 | 2.55E-06 | 33.75% |
| **Triglycerides\|\|ebi-a-GCST90092992** | 80.50436 | 63 | 0.067741 | 21.74% |
| **HDL cholesterol\|\|ebi-a-GCST90025956** | 505.2834 | 330 | 1.66E-09 | 34.69% |
| **LDL cholesterol\|\|ebi-a-GCST90018961** | 176.5466 | 145 | 0.038242 | 17.87% |
| **LDL cholesterol\|\|ebi-a-GCST90092814** | 51.49675 | 42 | 0.149508 | 18.44% |
| **Apolipoprotein A1 levels\|\|\|ebi-a-GCST90025955** | 404.6296 | 267 | 1.06E-07 | 34.01% |
| **Apolipoprotein B levels\|\|ebi-a-GCST90025952** | 209.6374 | 180 | 0.064459 | 14.14% |
| **Fasting glucose \|\| id:ebi-a-GCST90002232** | 65.1351 | 57 | 0.214703 | 12.49% |
| **Calcium levels\|\|ebi-a-GCST90025990** | 268.6631 | 214 | 0.006617 | 20.35% |

Q: Cochran Q test；Q_df: degrees of freedom of Q test; Q_pval: P valve of Q test
