# Supplementary material for: Circulating Metabolic Factors Mediating the Effect of Obesity‐Related Indicators on Meniscal Injuries: A Mendelian Randomization Study
Source: Int J Genomics. 2026 Feb 23;2026:8056288. doi: 10.1155/ijog/8056288 (PMC12929031; doi:10.1155/ijog/8056288)
Supplement: Supplementary file 22 — Supporting Information 22 Table S15: Pleiotropy test of MR analysis of circulating metabolic factors for meniscal injuries. [file IJOG-2026-8056288-s018.docx]

**Table S15. Pleiotropy test of MR analysis of circulating metabolic factors for meniscal injuries**

| **Exposure** | **MR-Egger intercept** | **Standard error** | **pval** |
| --- | --- | --- | --- |
| **uric acid \|\|ebi-a-GCST90018977** | 0.003713 | 0.002439 | 0.129 |
| **Bone mineral density\|\|ebi-a-GCST005348** | -0.001600 | 0.005901 | 0.787 |
| **Serum 25-Hydroxyvitamin D levels\|\|ebi-a-GCST90000618** | 0.004189 | 0.00253 | 0.101 |
| **TC\|\|ebi-a-GCST90025953** | -0.00178 | 0.001995 | 0.375 |
| **Triglycerides\|\|ebi-a-GCST90018975** | 0.000605 | 0.002119 | 0.776 |
| **Triglycerides\|\|ebi-a-GCST90092992** | 0.000989 | 0.000805 | 0.220 |
| **HDL cholesterol\|\|ebi-a-GCST90025956** | 0.000914 | 0.004078 | 0.823 |
| **LDL cholesterol\|\|ebi-a-GCST90018961** | -0.00169 | 0.002105 | 0.423 |
| **LDL cholesterol\|\|ebi-a-GCST90092814** | 0.001636 | 0.004817 | 0.736 |
| **Apolipoprotein A1 levels\|\|\|ebi-a-GCST90025955** | 0.000932 | 0.00179 | 0.603 |
| **Apolipoprotein B levels\|\|ebi-a-GCST90025952** | -0.00238 | 0.001661 | 0.153 |
| **Fasting glucose \|\| id:ebi-a-GCST90002232** | -0.00776 | 0.007613 | 0.312 |
| **Calcium levels\|\|ebi-a-GCST90025990** | 0.003626 | 0.002301 | 0.117 |
