# Supplementary material for: Circulating Metabolic Factors Mediating the Effect of Obesity‐Related Indicators on Meniscal Injuries: A Mendelian Randomization Study
Source: Int J Genomics. 2026 Feb 23;2026:8056288. doi: 10.1155/ijog/8056288 (PMC12929031; doi:10.1155/ijog/8056288)
Supplement: Supplementary file 24 — Supporting Information 24 Table S17: Heterogeneity test of MR of obesity‐related indicators for circulating metabolic indicators. [file IJOG-2026-8056288-s020.docx]

**Table S17. Heterogeneity test of MR of obesity-related indicators for circulating metabolic indicators**

| **Exposure** | **Outcome** | **Q** | **Q_df** | **Q_pval** | **I^2^（%）** |
| --- | --- | --- | --- | --- | --- |
| **Waist circumference\|\|ebi-a-GCST90014020** | uric acid \|\|ebi-a-GCST90018977 | 1009.46 | 293 | 0.00000 | 70.97% |
| **Waist circumference\|\|ebi-a-GCST90014020** | Bone mineral density\|\|ebi-a-GCST005348 | 592.976 | 302 | 0.00000 | 49.07% |
| **Waist circumference\|\|ebi-a-GCST90014020** | Serum 25-Hydroxyvitamin D levels\|\|ebi-a-GCST90000618 | 720.05 | 279 | 0.00000 | 61.25% |
| **Waist circumference\|\|ebi-a-GCST90014020** | TC\|\|ebi-a-GCST90025953 | 138.452 | 29 | 0.00000 | 79.05% |
| **Waist circumference\|\|ebi-a-GCST90014020** | HDL cholesterol\|\|ebi-a-GCST90025956 | 104.355 | 28 | 0.00000 | 73.17% |
| **Waist circumference\|\|ebi-a-GCST90014020** | LDL cholesterol\|\|ebi-a-GCST90092814 | 500.728 | 316 | 0.00000 | 36.89% |
| **Waist circumference\|\|ebi-a-GCST90014020** | Apolipoprotein A1 levels\|\|\|ebi-a-GCST90025955 | 107.053 | 29 | 0.00000 | 72.91% |
| **Waist circumference\|\|ebi-a-GCST90014020** | Apolipoprotein B levels\|\|ebi-a-GCST90025952 | 424.682 | 117 | 0.00000 | 72.45% |
| **Waist circumference\|\|ebi-a-GCST90014020** | Fasting glucose\|\| id:ebi-a-GCST90002232 | 586.297 | 297 | 0.00000 | 49.34% |
| **Waist circumference\|\|ebi-a-GCST90014020** | Calcium levels\|\|GCST90025990 | 408.167 | 117 | 0.00000 | 71.34% |
| **BMI\|\|ukb-b-2303** | Bone mineral density\|\|ebi-a-GCST005348 | 800.148 | 426 | 0.00000 | 46.76% |
| **BMI\|\|ukb-b-2303** | Serum 25-Hydroxyvitamin D levels\|\|ebi-a-GCST90000618 | 918.545 | 392 | 0.00000 | 57.32% |
| **BMI\|\|ukb-b-2303** | TC\|\|ebi-a-GCST90025953 | 130.927 | 50 | 0.00000 | 61.81% |
| **BMI\|\|ukb-b-2303** | HDL cholesterol\|\|ebi-a-GCST90025956 | 170.402 | 47 | 0.00000 | 72.42% |
| **BMI\|\|ukb-b-2303** | LDL cholesterol\|\|ebi-a-GCST90092814 | 664.336 | 428 | 0.00000 | 35.57% |
| **BMI\|\|ukb-b-2303** | Apolipoprotein A1 levels\|\|\|ebi-a-GCST90025955 | 112.83 | 48 | 0.00000 | 57.46% |
| **BMI\|\|ukb-b-2303** | Apolipoprotein B levels\|\|ebi-a-GCST90025952 | 143.801 | 51 | 0.00000 | 64.53% |
| **BMI\|\|ukb-b-2303** | Fasting glucose\|\| id:ebi-a-GCST90002232 | 693.229 | 420 | 0.00000 | 39.41% |
| **BMI\|\|ukb-b-2303** | Calcium levels\|\|GCST90025990 | 215.288 | 50 | 0.00000 | 76.78% |
| **Body fat percentage\|\|ebi-a-GCST90013975** | uric acid \|\|ebi-a-GCST90018977 | 1413.47 | 348 | 0.00000 | 75.38% |
| **Body fat percentage\|\|ebi-a-GCST90013975** | Bone mineral density\|\|ebi-a-GCST005348 | 747.345 | 360 | 0.00000 | 51.83% |
| **Body fat percentage\|\|ebi-a-GCST90013975** | Serum 25-Hydroxyvitamin D levels\|\|ebi-a-GCST90000618 | 807.04 | 327 | 0.00000 | 59.48% |
| **Body fat percentage\|\|ebi-a-GCST90013975** | TC\|\|ebi-a-GCST90025953 | 208.105 | 44 | 0.00000 | 78.86% |
| **Body fat percentage\|\|ebi-a-GCST90013975** | HDL cholesterol\|\|ebi-a-GCST90025956 | 135.414 | 39 | 0.00000 | 71.20% |
| **Body fat percentage\|\|ebi-a-GCST90013975** | LDL cholesterol\|\|ebi-a-GCST90092814 | 668.252 | 371 | 0.00000 | 44.48% |
| **Body fat percentage\|\|ebi-a-GCST90013975** | Apolipoprotein A1 levels\|\|\|ebi-a-GCST90025955 | 155.953 | 40 | 0.00000 | 74.35% |
| **Body fat percentage\|\|ebi-a-GCST90013975** | Apolipoprotein B levels\|\|ebi-a-GCST90025952 | 549.412 | 155 | 0.00000 | 71.79% |
| **Body fat percentage\|\|ebi-a-GCST90013975** | Fasting glucose\|\| id:ebi-a-GCST90002232 | 747.699 | 355 | 0.00000 | 52.52% |
| **Body fat percentage\|\|ebi-a-GCST90013975** | Calcium levels\|\|GCST90025990 | 132.584 | 46 | 0.00000 | 65.31% |
| **Body fat percentage\|\|ebi-a-GCST90013975** | uric acid \|\|ebi-a-GCST90018977 | 1221.24 | 343 | 0.00000 | 71.91% |
| **Body fat percentage\|\|ebi-a-GCST90013975** | Bone mineral density\|\|ebi-a-GCST005348 | 676.721 | 366 | 0.00000 | 45.92% |
| **Leg fat percentage(right)\|\|ukb-b-20531** | Serum 25-Hydroxyvitamin D levels\|\|ebi-a-GCST90000618 | 994.298 | 334 | 0.00000 | 66.41% |
| **Leg fat percentage(right)\|\|ukb-b-20531** | TC\|\|ebi-a-GCST90025953 | 152.328 | 33 | 0.00000 | 78.34% |
| **Leg fat percentage(right)\|\|ukb-b-20531** | HDL cholesterol\|\|ebi-a-GCST90025956 | 149.345 | 29 | 0.00000 | 80.58% |
| **Leg fat percentage(right)\|\|ukb-b-20531** | LDL cholesterol\|\|ebi-a-GCST90092814 | 650.734 | 364 | 0.00000 | 44.06% |
| **Leg fat percentage(right)\|\|ukb-b-20531** | Apolipoprotein A1 levels\|\|\|ebi-a-GCST90025955 | 126.092 | 31 | 0.00000 | 75.41% |
| **Leg fat percentage(right)\|\|ukb-b-20531** | Apolipoprotein B levels\|\|ebi-a-GCST90025952 | 197.697 | 35 | 0.00000 | 82.30% |
| **Leg fat percentage(right)\|\|ukb-b-20531** | Fasting glucose\|\| id:ebi-a-GCST90002232 | 671.843 | 359 | 0.00000 | 46.56% |
| **Leg fat percentage(right)\|\|ukb-b-20531** | Calcium levels\|\|GCST90025990 | 132.057 | 35 | 0.00000 | 73.50% |
| **Leg fat percentage(right)\|\|ukb-b-20531** | uric acid \|\|ebi-a-GCST90018977 | 1223.44 | 339 | 0.00000 | 72.29% |
| **Leg fat percentage(right)\|\|ukb-b-20531** | Bone mineral density\|\|ebi-a-GCST005348 | 710.69 | 362 | 0.00000 | 49.06% |
| **Leg fat percentage(left)\|\|ukb-b-18377** | Serum 25-Hydroxyvitamin D levels\|\|ebi-a-GCST90000618 | 921.415 | 331 | 0.00000 | 64.08% |
| **Leg fat percentage(left)\|\|ukb-b-18377** | HDL cholesterol\|\|ebi-a-GCST90025956 | 99.6082 | 30 | 0.00000 | 69.88% |
| **Leg fat percentage(left)\|\|ukb-b-18377** | LDL cholesterol\|\|ebi-a-GCST90092814 | 664.336 | 428 | 0.00000 | 35.57% |
| **Leg fat percentage(left)\|\|ukb-b-18377** | Apolipoprotein A1 levels\|\|\|ebi-a-GCST90025955 | 83.0073 | 32 | 0.00000 | 61.45% |
| **Leg fat percentage(left)\|\|ukb-b-18377** | Apolipoprotein B levels\|\|ebi-a-GCST90025952 | 219.111 | 36 | 0.00000 | 83.57% |
| **Leg fat percentage(left)\|\|ukb-b-18377** | Fasting glucose\|\| id:ebi-a-GCST90002232 | 721.761 | 357 | 0.00000 | 50.53% |
| **Leg fat percentage(left)\|\|ukb-b-18377** | Calcium levels\|\|GCST90025990 | 153.552 | 34 | 0.00000 | 77.86% |

Q: Cochran Q test；Q_df: degrees of freedom of Q test; Q_pval: P valve of Q test
