# Supplementary material for: Circulating Metabolic Factors Mediating the Effect of Obesity‐Related Indicators on Meniscal Injuries: A Mendelian Randomization Study
Source: Int J Genomics. 2026 Feb 23;2026:8056288. doi: 10.1155/ijog/8056288 (PMC12929031; doi:10.1155/ijog/8056288)
Supplement: Supplementary file 28 — Supporting Information 28 Table S21: Estimation of MR causal effects of obesity‐related indicators on circulating metabolic factors (IVW fixed‐effects model). [file IJOG-2026-8056288-s024.docx]

**Table S21.** Estimation of MR causal effects of obesity-related indicators on circulating metabolic factors (IVW fixed-effects model).

| **Exposure** | **outcome** | **Number of SNPs** | **β** | **Standard error** | **OR (95%CI)** | ***p*-value** |
| --- | --- | --- | --- | --- | --- | --- |
| **Waist circumference\|\|ebi-a-GCST90014020** | LDL cholesterol\|\|ebi-a-GCST90092814 | 318 | -0.12489 | 0.016462 | 0.8826(0.8546,0.9115) | 0.0000 |
| **BMI\|\|ukb-b-2303** | LDL cholesterol\|\|ebi-a-GCST90092814 | 430 | -0.10589 | 0.012524 | 0.8995(0.8777,0.9219) | 0.0000 |
| **Body fat percentage\|\|ebi-a-GCST90013975** | LDL cholesterol\|\|GCST90092814 | 373 | -0.11567 | 0.017936 | 0.8908(0.8599,0.9226) | 0.0000 |
| **Leg fat percentage(left)\|\|ukb-b-18377** | LDL cholesterol\|\|GCST90092814 | 363 | -0.09809 | 0.023137 | 0.9066(0.8664,0.9486) | 0.0000 |
| **Leg fat percentage(right)\|\|ukb-b-20531** | LDL cholesterol\|\|GCST90092814 | 366 | -0.12852 | 0.02285 | 0.8794(0.8408,0.9197) | 0.0000 |

SNP, single-nucleotide polymorphism; OR, odds ratio; CI, confidence interval.
