# Supplementary material for: Circulating Metabolic Factors Mediating the Effect of Obesity‐Related Indicators on Meniscal Injuries: A Mendelian Randomization Study
Source: Int J Genomics. 2026 Feb 23;2026:8056288. doi: 10.1155/ijog/8056288 (PMC12929031; doi:10.1155/ijog/8056288)
Supplement: Supplementary file 29 — Supporting Information 29 Table S22: Estimation of MR causal effects of obesity‐related indicators on circulating metabolic factors (IVW random‐effects model). [file IJOG-2026-8056288-s025.docx]

**Table S22**. Estimation of MR causal effects of obesity-related indicators on circulating metabolic factors (IVW random-effects model).

| **Exposure** | **outcome** | **Number of SNPs** | **β** | **Standard error** | **OR (95%CI)** | ***p*-value** |
| --- | --- | --- | --- | --- | --- | --- |
| Waist circumference\|\|ebi-a-GCST90014020 | uric acid \|\|ebi-a-GCST90018977 | 295 | 0.25135 | 0.01482 | 1.2858(1.2489,1.3237) | 0.0000 |
| Waist circumference\|\|ebi-a-GCST90014020 | HDL cholesterol\|\|ebi-a-GCST90025956 | 30 | -0.0989 | 0.0484 | 0.9058(0.8238,0.9959) | 0.0410 |
| BMI\|\|ukb-b-2303 | Serum 25-Hydroxyvitamin D levels\|\|ebi-a-GCST90000618 | 394 | 0.032227 | 0.01018 | 1.0321(1.012,1.0536) | 0.0015 |
| BMI\|\|ukb-b-2303 | Apolipoprotein B levels\|\|ebi-a-GCST90025952 | 53 | -0.0716 | 0.02734 | 0.9309(0.8823,0.9822) | 0.0088 |
| Body fat percentage\|\|ebi-a-GCST90013975 | uric acid \|\|ebi-a-GCST90018977 | 350 | 0.24539 | 0.01705 | 1.2781(1.2361,1.3215) | 0.0000 |
| Body fat percentage\|\|ebi-a-GCST90013975 | Apolipoprotein A1 levels\|\|\|ebi-a-GCST90025955 | 42 | 0.1366 | 0.04939 | 1.1464(1.0406,1.2629) | 0.0057 |
| Body fat percentage\|\|ebi-a-GCST90013975 | Apolipoprotein B levels\|\|ebi-a-GCST90025952 | 157 | -0.0659 | 0.02446 | 0.9362(0.8924,0.9822) | 0.0070 |
| Leg fat percentage(left)\|\|ukb-b-18377 | uric acid \|\|ebi-a-GCST90018977 | 341 | 0.32713 | 0.02074 | 1.3870(1.3317,1.4445) | 0.0000 |
| Leg fat percentage(left)\|\|ukb-b-18377 | Apolipoprotein A1 levels\|\|\|ebi-a-GCST90025955 | 34 | 0.13025 | 0.05679 | 1.1391(1.0191,1.2732) | 0.0218 |
| Leg fat percentage(right)\|\|ukb-b-20531 | uric acid \|\|ebi-a-GCST90018977 | 345 | 0.33058 | 0.02035 | 1.3918(1.3373,1.4484) | 0.0000 |
| Leg fat percentage(right)\|\|ukb-b-20531 | Serum 25-Hydroxyvitamin D levels\|\|ebi-a-GCST90000618 | 336 | 0.0475 | 0.0203 | 1.0486(1.0077,1.0912) | 0.0193 |

SNP, single-nucleotide polymorphism; OR, odds ratio; CI, confidence interval.
