# Supplementary material for: Circulating Metabolic Factors Mediating the Effect of Obesity‐Related Indicators on Meniscal Injuries: A Mendelian Randomization Study
Source: Int J Genomics. 2026 Feb 23;2026:8056288. doi: 10.1155/ijog/8056288 (PMC12929031; doi:10.1155/ijog/8056288)
Supplement: Supplementary file 30 — Supporting Information 30 Table S23: Sobel test of circulating metabolic indicators mediating effects of obesity‐related indicators on meniscal injuries. [file IJOG-2026-8056288-s026.docx]

**Table S23.** Sobel test of circulating metabolic indicators mediating effects of obesity-related indicators on meniscal injuries

| **Model** | **Z value** | ***P* value** |
| --- | --- | --- |
| **model1** | 0.876976 | 0.3805 |
| **model2** | 0.424786 | 0.670993 |
| **model3** | 0.224435 | 0.822418 |
| **Model4** | 1.220849 | 0.222143 |
| **Model5** | 0.052923 | 0.957794 |
| **Model6** | -0.62272 | 0.53347 |
| **Model7** | 1.983231 | 0.047342 |
| **Model8** | 0.43645 | 0.662511 |
| **Model9** | -0.76945 | 0.441629 |
| **Model10** | -0.6822 | 0.49511 |
| **Model11** | 1.758473 | 0.078667 |
| **model12** | 1.236028 | 0.216448 |
| **model13** | 1.340842 | 0.179972 |
| **Model14** | 0.699385 | 0.484311 |
| **Model15** | 0.205276 | 0.837356 |
| **Model16** | -1.02818 | 0.303865 |
