# Supplementary material for: Circulating Metabolic Factors Mediating the Effect of Obesity‐Related Indicators on Meniscal Injuries: A Mendelian Randomization Study
Source: Int J Genomics. 2026 Feb 23;2026:8056288. doi: 10.1155/ijog/8056288 (PMC12929031; doi:10.1155/ijog/8056288)
Supplement: Supplementary file 31 — Supporting Information 31 Table S24: Effect of circulating metabolic indicators mediating obesity‐related indicators on meniscal injuries through MR. [file IJOG-2026-8056288-s027.docx]

**Table S24.** Effect of circulating metabolic indicators mediating obesity-related indicators on meniscal injuries through MR.

| **Model** | **Exposure (E)** | **Mediator (M)** | **Direct effect E–M (95% CI)** | **Direct effect M–O (95% CI)** | **Total effect E–O** | **Direct effect E–O** | **Mediation effect (95% CI)** |
| --- | --- | --- | --- | --- | --- | --- | --- |
| **Model 7** | Body fat percentage\|\|ebi-a-GCST90013975 | uric acid \|\|ebi-a-GCST90018977 | 0.2453 (0.2119,0.2788) | 0.1283(0.0152,0.2414) | 0.4586 (0.3122,0.6051) | 0.4907(0.3202, 0.6612) | 0.0222(0.0002, 0.0442) |
